# Bioprospection of entomopathogenic fungi natives of Brazilian Semi-arid with potential for biological control of Insect-pests

**DOI:** 10.1007/s11274-026-04946-6

**Published:** 2026-04-18

**Authors:** Tárcio Souza Santos, Jackson Freitas de Almeida Santos, Emilly Lourdes Tavares Santos, Thomaz Soares Santos, Josefa Lívia Silva Leite, Arie Fitzgerald Blank, Marcelo da Costa Mendonça

**Affiliations:** 1 Federal University of Sergipe (UFS), Av. Marcelo Deda Chagas, s/n, São Cristóvão, 49100-000 Brazil; 2 Tiradentes University (UNIT), Industrial Biotechnology Postgraduate Av Murilo Dantas 300, Aracaju, 49032-490 Brazil; 3 Sergipe Agricultural Development Company (Emdagro), Av. Carlos Rodrigues da Cruz, s/n, Aracaju, 49081-015 Brazil

**Keywords:** *Beauveria bassiana*, Biological control of pests, Bioinsecticide, *Metarhizium pinghaense*, Prospection

## Abstract

Entomopathogenic fungi (EPF) are the most prospected and used microorganisms for agricultural pest control worldwide. In this context, the semi-arid region of Brazil is a promising location for the collection of new EPF, due to its extreme temperature, radiation and precipitation characteristics, which can favor the adaptation of native microorganisms to these stress factors. However, there is no previous report of prospecting EPF isolates in the semi-arid region of the state of Sergipe/Brazil. Therefore, this study aimed to prospect EPF from the semi-arid region of the state of Sergipe/Brazil, evaluate their potential application in the biological control of agricultural insect pests, and their tolerance to diverse types of stress. Fourteen fungal isolates were obtained, but only six isolates showed high pathogenicity, according to Koch’s Postulates. The isolates were identified as *Beauveria bassiana* (five isolates) and *Metarhizium pinghaense* (one isolate), by molecular analysis. The fungi showed intraspecific and interspecific variations in the production of aerial conidia in rice, enzymatic production, and in their tolerance to the diverse types of stress tested. Thus, the semi-arid region of the state of Sergipe/Brazil is promising for obtaining new isolates of EPF with high potential for use in agricultural pest control.

**Graphical Abstract:**

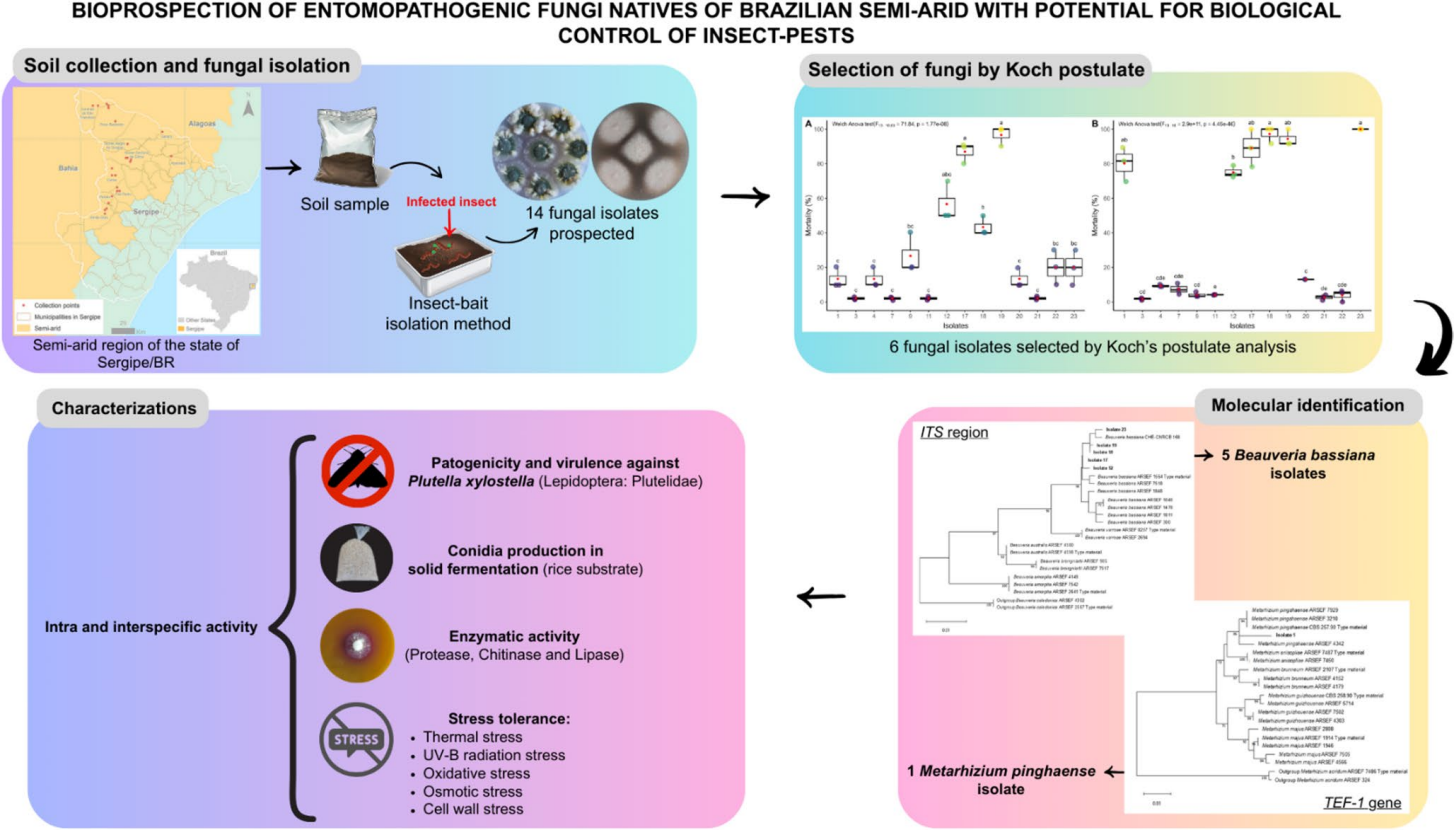

**Supplementary Information:**

The online version contains supplementary material available at 10.1007/s11274-026-04946-6.

## Introduction

The global concern about the sustainability of agricultural production for food production has favored the popularization of biological methods of agricultural pest control (Basnet et al. 2023; Deguine et al. 2023; Parra 2023), due to the insects were the main cause of damage in agricultural plantations (Oliveira et al. 2013; Adelino et al. 2021). In this regard, EPF are the most used biological agents for insect control, as they are microorganisms specialized in infecting the bodies of invertebrate arthropods, with an emphasis on insects, causing diseases that can lead to their death. These fungi are commonly found in plants (endophytic colonization), water, and soil, which is considered a natural reservoir of various microbial species and widely used for collecting and isolating new strains (Litwin et al. 2020; Rossouw et al. 2023; Sharma et al. 2023).

Different species or isolates of EPF may exhibit variations in some of their biological characteristics (such as pathogenicity, virulence, conidia production, and stress tolerance), generally caused by the influence of biotic factors (insects, plants, and competition for nutrients with other microorganisms) and abiotic factors (temperature, humidity, and radiation) present in the native habitat of the fungi (Shin et al. 2017; Acheampong et al. 2020; Licona-Juárez et al. 2023). These biotic and abiotic factors can affect the expression of genes associated with these characteristics, such as the NRPS (non-ribosomal peptides synthetases) and PKS genes (polyketides synthetases) and other genes associated to virulent factors (such as proteases, lipases, and chitinases), (Pedrini 2022; Vidhate et al. 2022), and laccase production genes, related to increased conidiation, tolerance to abiotic stress, and pathogenicity (Wu et al. 2025). Therefore, the environmental characteristics of the area used to prospect EPF can favor the obtaining of isolates that are more resistant to these abiotic factors (Dias et al. 2021).

Studies on the prospecting of EPF in Brazil have been mainly directed towards evaluating the presence of these microorganisms in biomes such as the Cerrado and the Atlantic Forest or are related to the prospecting of EPF in regions with commercially significant agricultural crops, such as sugarcane plantations (Mascarin et al. 2019; Souza et al. 2020; Rocha et al. 2022). Therefore, areas explored little for this purpose, such as the semi-arid region, stand out as promising environments for the search for new microorganisms. The semi-arid region of Brazil is located mainly in the northeast of the country and is composed of the Caatinga biome, which is predominantly herbaceous and grassy vegetation, with the presence of trees and shrubs in lower density. Additionally, it has rugged terrain, shallow soil, low rainfall (500–700 mm of rain/year), high temperature, and low humidity (Prata 2013).

The semi-arid region of Brazil is known for harboring a great diversity of animals, plants, and microorganisms with potential for application in various areas, including insect control. However, the resources contained in this habitat need to be better known and explored to make the most of their biotechnological potential (Lopes et al. 2016; Carvalho et al. 2023; Silva and Siqueira 2023). Therefore, this study aimed to prospect for entomopathogenic soil fungi in the semi-arid region of Brazil using the insect-bait method with *Tenebrio molitor* (L.) (Coleoptera: Tenebrionidae) identify them through molecular analysis and to characterize its pathogenicity and virulence against pest insect *Plutella xylostella* (L.) (Lepidoptera: Plutellidae), conidia production in rice, hydrolytic enzyme production (protease, chitinase and lipase), and tolerance to different types of stress in order to elucidate its potential application in the biological control of pests.

## Materials and methods

### Insect rearing

The insects *T. molitor* (Coleoptera: Tenebrionidae) (the model insect used in the isolation of fungi), and *P. xylostella* (Lepidoptera: Plutellidae) (used in pathogenicity assays as the target pest insect of the work), were reared at the Laboratory of Biotechnological Pest Control - LCBiotec (SergipeTec - Sergipe Technology Park, São Cristóvão, SE). *T. molitor* larvae were reared in plastic containers with wheat bran (25 ± 2 °C, RH 60 ± 10%, 12 h photophase). *P. xylostella* caterpillars were reared in plastic containers (16 by 23.5 by 36 cm) with organic cabbage leaves provided as a food source. All insects were kept in a climate-controlled room at 26 ± 2 °C, 60 ± 10% relative humidity and a 12 h photophase.

### Study area and soil sampling

**Fig. 1:**
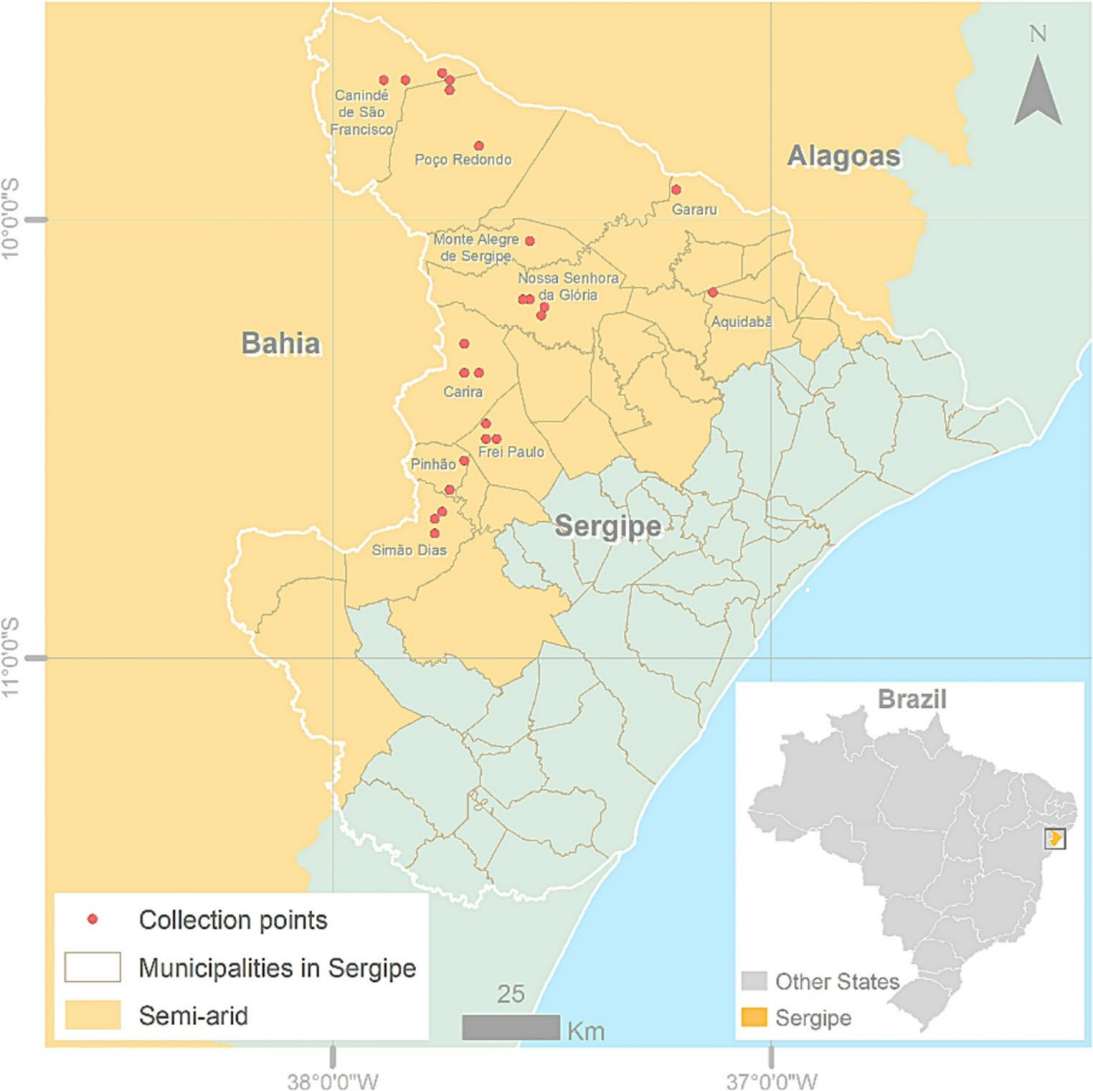
Map of the state of Sergipe, Brazil. The semi-arid region is highlighted in orange. The red dots indicate soil collection points within the municipalities selected for prospecting for entomopathogenic fungi. Illustration made in ArcGis software version 10.6

The collection area for EPF was realized in 10 municipalities of the semi-arid region of the state of Sergipe: Aquidabã, Canindé do São Francisco, Carira, Frei Paulo, Gararu, Monte Alegre, Nossa Senhora da Glória, Pinhão, Poço Redondo and Simão Dias (Fig. 1). All collection points were marked based on their geographical location with GPS equipment (Garmin, ETREX 10) and characterized based on the type of existing vegetation (Table S1). For soil sampling, the surface vegetation was removed and three random soil sub-samples were collected from each point at a depth of 20 cm with 1 m of distance between each sub-sample. The soil sub-samples were then mixed to form a composite sample of 500 g of soil, placed in sterile plastic bags and transported to the laboratory, where they were stored at 4 °C until processing.

### Isolation of entomopathogenic fungi

The isolation of EPF was conducted using the insect-bait method (Zimmermann 1986), utilizing larvae of *T. molitor*, insect widely used for isolating EPF in soil samples (Kim et al. 2018; Masoudi et al. 2020; Domingues et al. 2022). The soil samples were dry at 25 ± 2 °C for 3 days, to reduce the presence of entomopathogenic nematodes. Subsequently, 100 g of soil was transferred into 250 mL plastic pots, moistened with 10 mL of sterile distilled water, and inoculated with 10 larvae of *T. molitor* (third instar). The pots were covered with *voile* fabric and stored in a climate-controlled environment (25 ± 2 °C, 12 h photophase) for 10 days. The dead larvae were washed in 70% (v/v) ethyl alcohol and sterile distilled water and then transferred to sterile Petri dishes (35 by 10 mm) lined with moistened filter paper (humid chamber), to stimulate the sporulation of the fungal hyphae and confirm the death of the insect by the action of the fungus (Noah et al. 2022). The plates were sealed and incubated in a B.O.D. incubator (Bio-Oxygen Demand incubator) (Solab, SL-225, Piracicaba, SP, Brazil), at 25 ± 2 °C and 12 h photophase.

Fungal isolates were obtained from *T. molitor* larvae in the presence of fungi by removing hyphae and subculturing them on Potato Dextrose Agar (PDA) (Kasvi, Pinhais, SP, Brazil) medium containing 0.25 g/L chloramphenicol (Sigma-Aldrich, St. Louis, USA) and 0.20 g/L tetracycline (Êxodo Científica, Sumaré, SP, Brazil). The fungal isolates were purified and then examined microscopically to assess the characteristics of their conidiophores and compare them with the description by Humber et al. 2012. Pure cultures of EPF were preserved at −20 °C in 10% (v/v) glycerol (Êxodo Científica, Sumaré, SP, Brazil).

### Preliminary evaluation of the pathogenicity of entomopathogenic fungi

A preliminary pathogenicity bioassay was conducted to validate the fungi’s entomopathogenic activity according to Koch’s postulates. All EPF obtained in the isolation step were cultured in Petri dishes with PDA medium supplemented with 0.25 g/L chloramphenicol and incubated in a B.O.D. incubator (25 ± 2 °C, 12 h photophase), for 7 days. The surface fungus was scraped with a metal spatula and homogenized in 0.05% (v/v) Tween 80^®^ solution (Êxodo Científica, Sumaré, SP, Brazil). The suspension was filtered through sterile gauze to separate fungal mycelium, and the concentration of conidia was determined using a Neubauer chamber under a 400× optical microscope. The concentration of the fungal suspensions was subsequently adjusted to 5 × 10⁶ conidia/mL and 1 × 10⁷ conidia/mL. Conidial viability was assessed before applying the suspension in the bioassays. All fungi showed conidial viability > 90%.

In this preliminary pathogenicity assessment, all prospective EPF were applied to *T. molitor* (model insect used in the isolation step) (Kim et al. 2018; Kamarudin et al. 2022) and *P.* *xylostella* (key pest of cruciferous plants due to the significant damage caused to crops and its high resistance to chemical insecticides) (Shehzad et al. 2023; Michigan State University, 2024) to confirm pathogenicity and evaluate the occurrence of selectivity in the fungal isolates obtained. For the pathogenicity bioassay on *T. molitor*, third instar larvae were submerged in 20 mL of the fungal suspension (concentration of 5 × 10^6^ conidia/mL) for 5 s and transferred to a glass flask (110 by 68 mm) containing a 5 cm diameter disc of qualitative filter paper (Nalgon, Itupeva, SP, Brazil) and a piece of carrot to feed the larvae. The flasks were covered with *voile* fabric and kept in an air-conditioned room (25 ± 2 °C, 12 h photophase). Insect mortality was assessed every three days during the 15-day period (Santos et al. 2021).

The pathogenicity bioassay on *P. xylostella* was conducted using the residual effect evaluation method. For this, 8 cm diameter discs of organic cabbage leaves (*Brassica oleracea* L.) were dipped in the fungal suspension (1 × 10^7^ conidia/mL) and then maintained at room temperature (26 ± 2 °C) until the leaf surface dried. Subsequently, the cabbage discs were transferred to Petri dishes (90 by 10 mm), where the *P. xylostella* caterpillars (1st instar) were accommodated. The Petri dishes were sealed and stored in an air-conditioned room (26 ± 2 °C, RH 60 ± 10%, 12 h photophase). New cabbage discs (untreated) were offered for feeding the larvae on the third and sixth days of incubation. The accumulated mortality of the larvae was evaluated on the third and seventh days after the beginning of the experiment (Santos et al. 2022).

In both bioassays, insects that died during pathogenicity bioassays were rinsed with 70% (v/v) ethyl alcohol (Êxodo Científica, Sumaré, SP, Brazil), then washed with distilled water, and subsequently moved to a humid chamber. Each isolate of EPF was considered as a treatment and the application of 0.05% (v/v) sterile Tween 80^®^ solution, vehicle used in the preparation of fungal suspensions, was used as a control. The experiment was performed once. All treatments were analyzed in triplicate, with 15 insects per replicate.

### Molecular identification

DNA was extracted from fresh fungal mycelium using CTAB reagent (Neon, Suzano, SP, Brazil), following the method described by Dhar et al. 2019. Genomic DNA was subjected to polymerase chain reaction (PCR) for amplification of the *ITS* (Internal transcribed spacer) region, with the primers ITS1 and ITS4 (White et al. 1990), ribosomal gene of the translation elongation factor (*TEF-1*), with the primers EF1-728 F and EF1-986R (Carbone and Kohn 1999) and Beta-tubulin gene (*B-TUB*), with the primers TUB2Fd and TUB4Rd (Aveskamp et al. 2009).

The PCR reaction was performed using primers at a final concentration of 0.2 µM, dNTPS at 0.2 mM and 1 U of the GoTaq^®^ Green enzyme (Promega, Madison, WI, USA), in a final volume of 25 µL. The DNA amplification of each of the genes analyzed was performed following the parameters described in their reference articles, cited previously. Amplified products were verified using electrophoresis in 0.8% (w/v) agarose gel (Kasvi, Pinhais, SP, Brazil), followed by staining with ethidium bromide (Sigma-Aldrich, St. Louis, USA). The amplified products were purified by precipitation with polyethylene glycol (Labsynth, Diadema, SP, Brazil) (Schmitz and Riesner 2006) and submitted to the sequencing reaction by the chain termination method, using the Big Dye 3.1 reagent (Applied Biosystems, Massachusetts, EUA), followed by analysis in an automatic capillary sequencer (Applied Biosystems, Model: 3500 XL, Massachusetts, EUA).

For the molecular analysis of *Beauveria* isolates, the *ITS* region and the *B-TUB* gene were sequenced, while for the *Metarhizium* isolate, the *TEF-1* gene and the *B-TUB* gene were sequenced. These regions were chosen for molecular analysis because they are conserved regions of DNA with extensive use in the identification of EPF (Castro-Vásquez et al. 2020; Al-Qadi et al. 2022; Rajab et al. 2024). The sequences were compared to those available in the NCBI (National Center for Biotechnology Information – www.ncbi.nlm.nih) database using the Blastn tool to confirm the identification of the isolates. For the construction of the phylogenetic tree, sequences from the *ITS* region were used for the *Beauveria* isolates and sequences from the *TEF-1* gene for the *Metarhizium* isolate. The sequences were aligned by Clustal W with sequences of type and non-type strains obtained from scientific literature: *ITS* region (Rehner et al. 2011; Serna-Domínguez et al. 2018) and *TEF-1* (Bischoff et al. 2009) The reconstruction model with the best fit was based on Bayesian Information Criterion (BIC) analysis, using the ModelFinder tool (option “-m MF”) in the IQ-Tree software v. 3.0.1 (Kalyaanamoorthy et al. 2017). The Hasegawa-Kishino-Yano model with invariants sites (HKY + I) was the best model for *ITS* and *TEF-1* reconstruction. Consensus sequences were used to build a phylogenetic tree via the Maximum Likelihood method (1,000 replicates), using software MEGA version 11 (Tamura et al. 2021).

### Confirmation of pathogenicity and virulence of selected isolates of entomopathogenic fungi

Six EPF isolates, chosen from the initial pathogenicity test, were used in a second evaluation of insecticidal potential with *P. xylostella* larvae (1st instar). The EPF were cultured in Petri dishes with PDA medium supplemented with 0.25 g/L chloramphenicol and incubated in a B.O.D. incubator (25 ± 2 °C, 12 h photophase), for 7 days. The surface fungus was scraped with a metal spatula and homogenized in 0.05% (v/v) Tween 80^®^ solution (Êxodo Científica, Sumaré, SP, Brazil). The suspension was filtered through sterile gauze to separate fungal mycelium, and the concentration of conidia was determined using a Neubauer chamber under a 400× optical microscope. The concentration of the fungal suspensions was subsequently adjusted to 1 × 10⁷ conidia/mL. The fungi exhibited conidial viability > 90% before their application in the bioassay.

The bioassay was conducted following the protocol described in the preliminary pathogenicity bioassay. Larval mortality was monitored daily for 7 days, and deceased specimens were moved to a humid chamber to verify that the death was caused by fungal activity. The LT_50_ of EPF isolates, time required to cause death in 50% of treated insects, was measured to assess their level of virulence. Each entomopathogenic fungus isolate served as a treatment, with 0.05% (v/v) sterile Tween 80^®^ solution as the control. All treatments consisted of 4 replications, with 20 insects in each replication.

### Evaluation of aerial conidia production in rice substrate

Fungal suspensions of all EPF isolates (concentration = 1 × 10⁸ conidia/mL) were prepared according to the preliminary bioassay for evaluating fungal pathogenicity. 5 mL of conidia suspension were added to a 250 mL Erlenmeyer flask containing 50 g of sterile parboiled rice. The flasks were kept in a B.O.D. chamber (25 ± 2 °C, 12 h photophase) for 7 days. For quantified aerial conidia production, 1 g of rice containing fungus was stirred in 10 mL of 0.05% (v/v) Tween 80^®^ and the suspension produced was serially diluted and the conidia concentration was estimated for counting under an optical microscope (400×) using a Neubauer chamber.

The conidia produced were also evaluated for viability by inoculating 150 µL of the conidia suspension (10^6^ conidia/mL) into a Rodac Petri dish containing PDA medium. The plates were incubated in B.O.D. incubator (25 ± 2 °C, 12 h photophase), for 16–18 h. Conidial viability was determined by the percentage of germinated conidia present on the surface of the culture medium, observed by counting under an optical microscope (400×), with the total count limited to 100 conidia per plate. The analysis was conducted with 3 replicates per treatment.

### Production of insect Cuticle-degrading enzymes

The EPF isolates were grown in PDA culture medium (25 ± 2 °C, 12 h photophase) for 7 days. 6 mm diameter culture medium discs containing the fungus grown on their surface were transferred to the center of plastic Petri dishes (90 × 10 mm) containing culture media with enzyme activity inducers: skim milk (protease), olive oil (lipase), and chitin (chitinase). The media were prepared according to Lechuga et al. (2016). Evaluations were performed in triplicate, and all plates were incubated in B.O.D. (25 ± 2 °C, 12 h photophase) for 7 days. Lipase activity was evaluated by the presence and intensity of orange coloration in the fungal colonies after exposure to UV light (350 nm). A lipolytic activity index, ranging from 1 to 4, was assigned to the result of the lipolytic activity of each fungus, depending on the intensity of the orange color observed in its colonies, where: 1 = low activity, 2 = moderate activity, 3 = good activity and 4 = excellent activity. The enzymatic activity of protease and chitinase was evaluated based on the enzymatic activity index, where: ≤ 1.5 = low activity, 1.5–2 = moderate activity, 2–3 = good activity, > 3 = excellent activity. by formula:

$$\:Enzymatic\:activity\:index=\:\frac{inducer\:degradation\:zone}{colony\:diameter}$$

### Evaluation of the tolerance of isolates of entomopathogenic fungi to different stress

#### Heat stress

A fungal suspension with standardized concentration (1 × 10^2^ conidia/mL) was produced, according to the method described above. Twenty mL of the conidia suspension was transferred to 50 mL Erlenmeyers and placed in a rotary shaker (Solab, SL-223, Piracicaba, SP, Brazil), at 50 rpm, in the temperatures of 25 ± 2 °C, 35 ± 2 °C and 40 ± 2 °C for 2, 4, 6, 8, 10 and 12 h of exposure. Then, 100 µL of the suspension was inoculated, with Drigalski loop, in Petri dishes (57.7 by 12 mm) containing PDA culture medium. The plates were incubated in a B.O.D. incubator (25 ± 2 °C, 12 h photophase), for 5 days.

The experiments at a temperature of 45 ± 2 °C were performed using variable exposure times for each isolate, due to the existence of differences in their thermal tolerance. Therefore, the exposure times used were: isolate 1 (2, 4, 6, 8, 10, 12 h), isolate 12 (0.5, 1, 1.5, 2, 2.5, 3 and 3.5 h), isolate 18 (0.5, 1, 1.5, 2, 2.5 and 3 h), isolate 17 (1, 2, 3, 4, 5 and 6 h), isolate 23 (2, 4, 6 and 8 h) and the isolate 19 (1, 2, 3, 4 and 5 h). The temperatures used were selected based on previous tests evaluating the tolerance of EPF to hot climates and their tolerance to extreme thermal shock (Gava et al. 2022; Bernardo et al. 2020). Plating and incubation at 45°C followed the same parameters used for the other temperatures evaluated. After the incubation period, the number of colonies in the plate was evaluated to determine the number of colonies forming units (CFU/mL), by formula:

$$\:CFU\:=number\:of\:colonies\:\times\:\:dilution\:factor\:\times\:\:10\:$$

Fungal suspension without exposure to temperatures was used as control treatment. CFU assessments were performed with three replicates and data were normalized by control viability to calculate the relative viability percentage. The viability of fungal conidia over time of exposure to different temperatures was used to calculate the estimated effective time (ET) required for the inactivation of 50% and 90% of the treated conidia (Bernardo et al. 2020).

### UV-B Radiation stress

Rodac Petri dishes, containing 5 mL of PDA medium supplemented with 0.25 g/L chloramphenicol, were inoculated over their entire surface with 100 µL of the fungal suspension (1 × 10^2^ conidia/mL), and transferred to a radiation chamber composed of a wooden box (68 by 43 by 36 cm), containing a UV-B lamp attached to its lid (Philips, TL 20 W/01-RS, peak 311 nm). Fungi were exposed to UV-B light for different exposure times (30, 60, 90, 120, 150, 180 and 210 min), corresponding to a dose of 1.4, 2.8, 4.2, 5.6, 7.0, 8.2 and 9.8 kJ.m^− 2^ of UV-B radiation, according to the measurement made with a radiometer (Delta Ohm, HD 2302.0 Lightmeter, LP471 UVB probe), discounting the radiation loss caused by the Rodac plate covering.

After exposure, the plates were stored in a B.O.D. incubator (25 ± 2 °C, 12 h photophase) for 5 days. Evaluations were performed with three replicates, and fungal viability was transformed into a percentage of relative conidial viability, normalizing treatment results by the viability of the control (not exposed to radiation). The viability of fungal conidia over time of exposure to different doses of UV-B radiation was used to calculate the estimated effective dose (ED) required for the inactivation of 50% and 90% of the treated conidia (Bernardo et al. 2020).

### Fungal growth with oxidative stress, osmotic stress and cell wall stress inducers

Six millimeter diameter PDA culture medium discs, containing fungi grown for 7 days, were placed in the center of Petri dishes containing PDA without supplementation (control) or supplemented with different stress inductors: 2 mM Oxygen peroxide (H_2_O_2_), for oxidative stress, 1 M Sodium chloride (NaCl), for osmotic stress, and 0.3 mg/mL Congo Red dye, for cell wall stress (Xie et al. 2019). The plates were incubated in B.O.D. (25 ± 2 °C, 12 h photophase) for 7 days. After the incubation period, the colony diameter was measured with a caliper. The evaluation was performed in triplicate.

### Statistical analysis

In all bioassays of pathogenicity and virulence of EPF against insects, the mortality data of the treatments were corrected for control mortality using Abbott’s formula (Abbott 1925). Initially, all data were checked for normality and homogeneity of variances using the Shapiro-Wilk test and Levene’s test, respectively. Parametric data with homogeneous variances were analyzed using classical ANOVA, followed by Duncan’s post-hoc test (*p* < 0.05) (bioassay data confirming the pathogenicity and virulence of the selected isolates, conidia production, and fungal growth in the presence of stress inducers). Parametric data with non-homogeneous variances were analyzed using Welch’s ANOVA, followed by Games-Howell’s post-hoc test (*p* < 0.05) (data from the preliminary evaluation of the pathogenicity of prospected EPF).

The daily mortality of *P. xylostella* in the pathogenicity and virulence confirmation assay of the selected EPF was used to construct the survival curve using the Kaplan-Meier (Mantel-Cox) method to estimate the LT_50_ of the isolates, a method widely used for this purpose (Santos et al. 2021; García et al. 2022; Gava et al. 2022). Viability of fungal conidia over time of exposure to different temperatures and doses of UV-B radiation was fitted to a two-parameter Weibull model (Lima et al. 2024) to estimate the effective time (ET) and effective dose (ED) required for the inactivation of 50% and 90% of the treated conidia, respectively, using the function:

$$\:f\left(x\right)=\mathrm{exp}(-\mathrm{exp}\left(b\left(\mathrm{log}\left(x\right)-e\right)\right))$$

All analyses were performed using R software, version 4.4.3. (R Core Team 2025) and RStudio, version 2025.05.1. (Posit Team 2025) using different packages: “drc” (Ritz et al. 2015) and “ec50estimator” (Alves 2022) for modeling using the Weibull function, “survminer” (Kassambara et al. 2025) for survival analysis, “rstatix” (Kassambara 2022) for normality analysis (Shapiro-Wilk’s test), analysis of homogeneity of variances (Levene’s test), ANOVA and Welch’s ANOVA, and “DescTools” (Signorell et al., 2024) for Duncan’s posthoc test.

## Results

### Isolation of fungi and preliminary pathogenicity bioassay

**Fig. 2:**
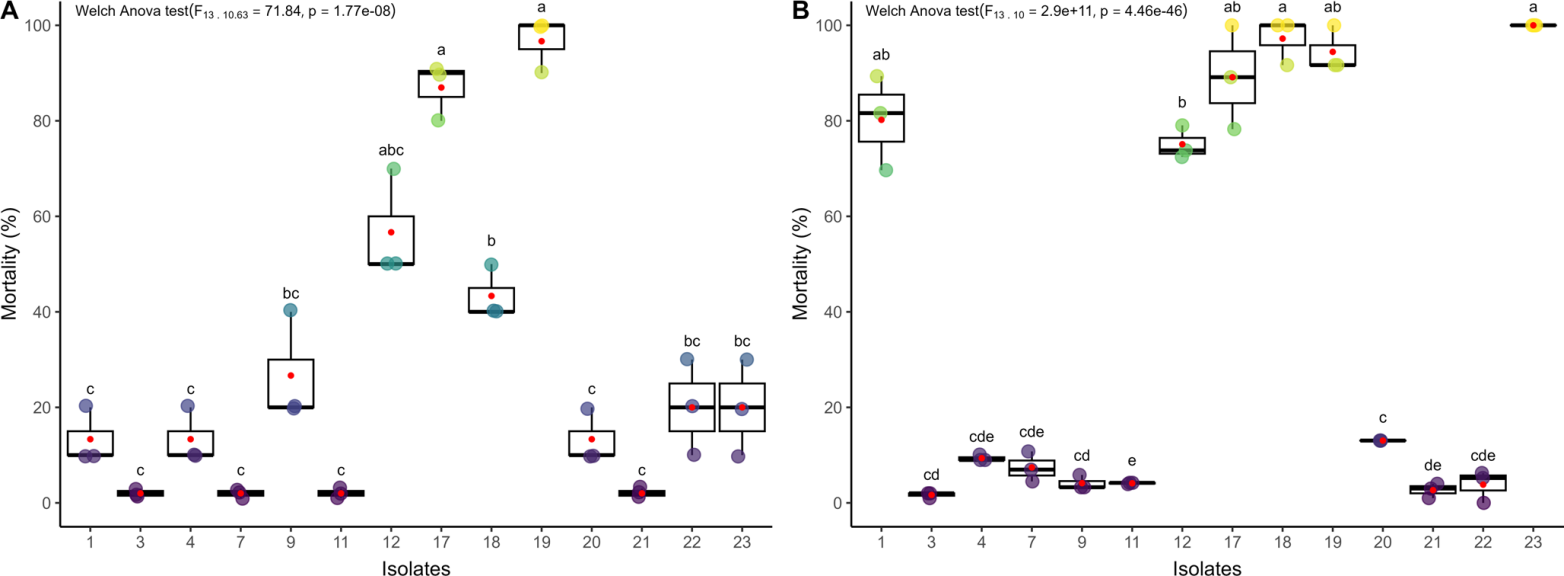
Pathogenicity of entomopathogenic fungal isolates against *Tenebrio molitor* (**A**) and *Plutella xylostella* (**B**). Isolates with the same letters do not differ significantly according to Games-Howell’s test (*p* < 0.05)

Soil samples allowed the collection of 14 fungal isolates (58.3% of positive samples) from 7 different municipalities: 2 isolates from the municipality of Frei Paulo (isolate 1 and 3), 1 isolate from the municipality of Carira (isolate 4), 1 isolate from the municipality of Pinhão (isolate 7), 2 isolates from the municipality of Simão Dias (isolate 9 and 11), 3 isolates from the municipality of Canindé de São Francisco (isolate 12, 21 and 22), 4 isolates from the municipality of Nossa Senhora da Glória (isolate 17, 18, 19 and 20), and 1 isolate from the municipality of Gararu (isolate 23). The *T. molitor* larvae used as bait in soil samples from the cities of Aquidabã, Monte Alegre, and Poço Redondo did not show fungal infection. Of the 14 isolates initially obtained, only 2 isolates (isolate 17 and isolate 19 = 14.28% of the prospected isolates) showed high pathogenicity (> 80%) on *T. molitor* larvae (Fig. 2A). When applied to *P. xylostella*, only 6 isolates (isolates 1, 12, 17, 18, 19 and 23 = 42.85% of the prospected isolates) promoted significant mortality in the treated larvae. Isolates 1, 17, 18, 19, and 23 showed high pathogenicity (> 80%), and isolate 12 showed moderate pathogenicity (60 ~ 80%), according to the scale proposed by Chang et al. (2021). (Fig. 2B).

### Molecular identification

**Fig. 3:**
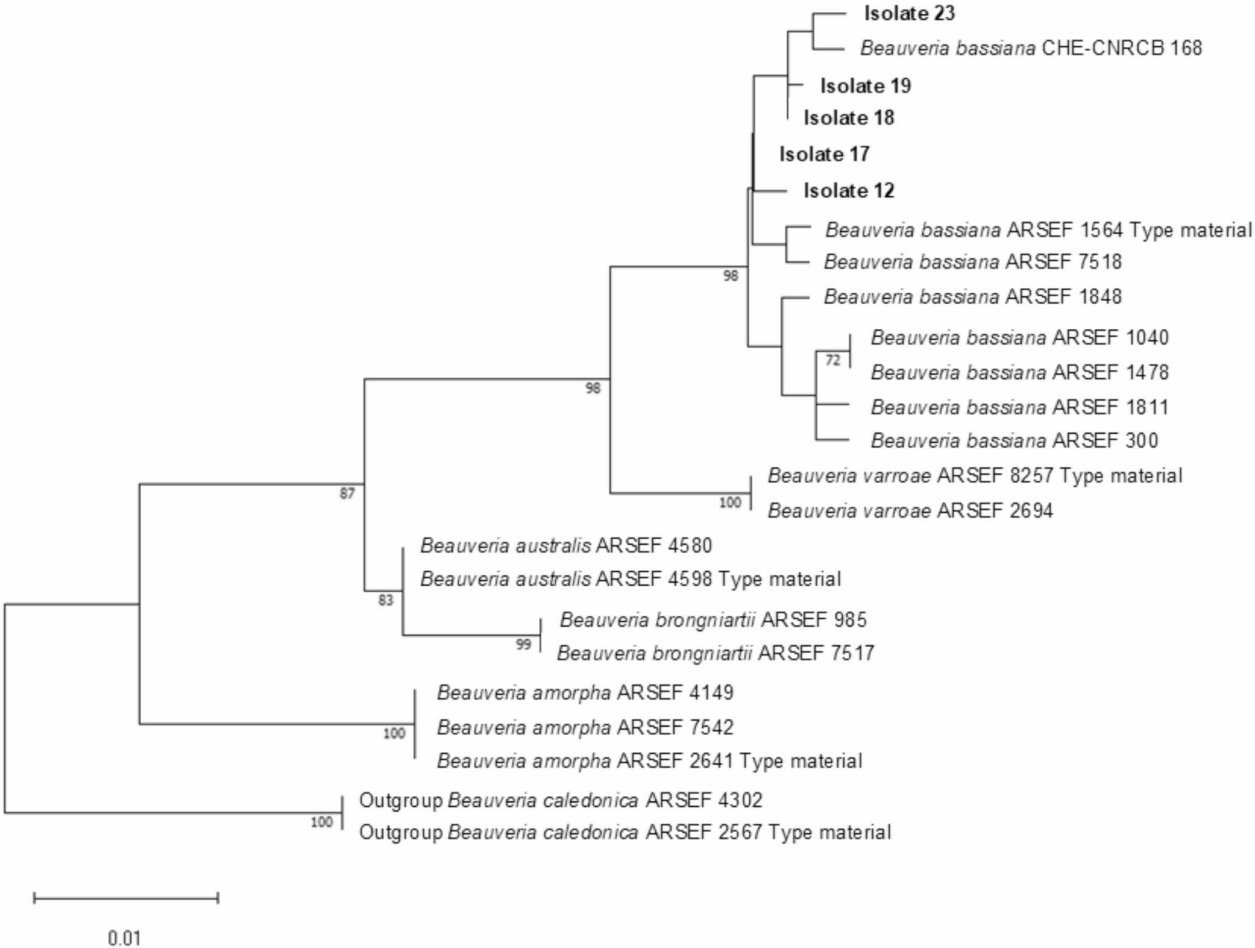
Maximum Likelihood Tree of the Intergenic Region *(ITS*) of *Beauveria bassiana* isolates

**Fig. 4:**
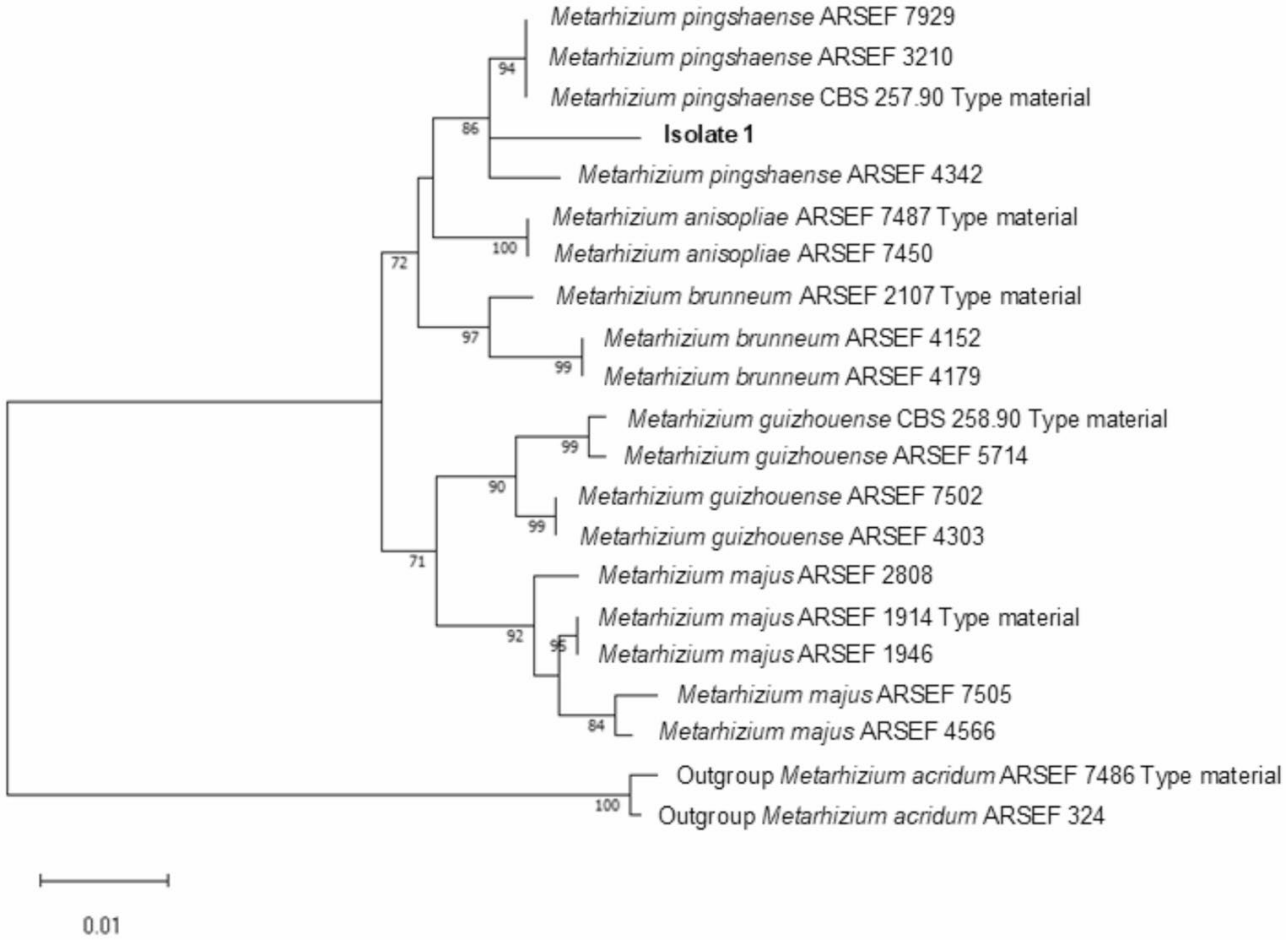
Maximum Likelihood Tree of the Translation Elongation Factor gene (*TEF-1*) of the *Metarhizium pinghaense* isolate

The genomic DNA sequences obtained in our study by amplifying and sequencing the *ITS* region, *TEF-1* and *B-TUB* genes of EPF exhibited homology with the species *Beauveria bassiana* (isolates 12, 17, 18, 19, and 23) and *Metarhizium pinghaense* (isolate 1) when compared with the nucleotide sequences present in the NCBI database using Blastn (Table S2). The phylogenetic analysis performed using the Maximum Likelihood method confirmed the identification suggested in the analysis using the Blastn tool (Figs. 3 and 4).

The identified EPF were deposited in the Entomopathogenic Fungi Bank of the Laboratory of Biotechnological Pest Control (LCBiotec) – Sergipe Agricultural Development Company - Emdagro (São Cristóvão/Sergipe/Brazil) and in the Collection of Microorganisms that are Biological Pest Control Agents in Agriculture/Invertebrate Fungi – Brazilian Agricultural Research Corporation – Embrapa Genetic Resources and Biotechnology (Brasília/DF/Brazil). All sequences obtained during molecular identification were deposited in the database (GenBank) of the National Center for Biotechnology Information – NCBI. (Table S3). The codes of the sequences collected in GenBank for the construction of phylogenetic trees are presented in Table S4.

### Confirmation of pathogenicity and virulence of selected isolates of entomopathogenic fungi

**Fig. 5:**
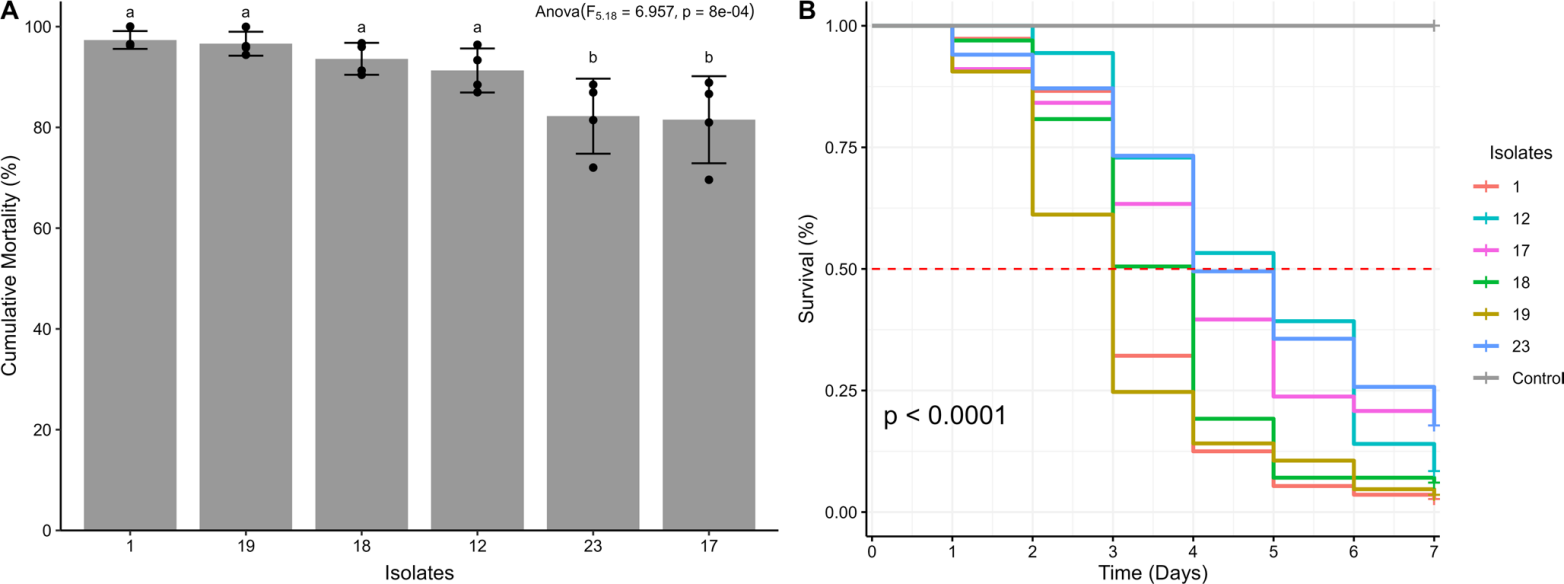
Evaluation of the pathogenicity and virulence of entomopathogenic fungal isolates on *Plutella xylostella*. **A** = Pathogenicity of entomopathogenic fungal isolates, expressed as the cumulative mortality of treated insects. Isolates with bars (± standard deviation) with the same letters do not differ significantly according to Duncan’s test (*p* < 0.05). **B** = Survival curve, constructed by the Kaplan-Meier method, with daily mortality data for *Plutella xylostella* treated with entomopathogenic fungal isolates. The dashed red line in the survival curve indicates the LT_50_ of the isolates

The pathogenicity of EPF isolates against *P. xylostella* larvae present significant difference (F_5.18_ = 6.957, *p* = 8 × 10^− 4^). The isolates 1 (*M. pinghaense*), 19 (*B. bassiana*), 18 (*B. bassiana*) and 12 (*B. bassiana*) promoted the highest mortality rates on *P. xylostella* (> 90%) and did not differ from each other, according to Duncan’s test (Fig. 5A). The isolates 23 (*B. bassiana*) and 17 (*B. bassiana*) exhibited pathogenicity rates ≤ 82%. Isolates 19 (*B. bassiana*) and 1 (*M. pinghaense*) showed the lowest estimated values for the median LT_50_ (3 days), being, therefore, the most virulent among all the isolates tested. The other isolates presented LT_50_ between 4 and 5 days (Fig. 5B).

### Evaluation of aerial conidia production in rice substrate

**Fig. 6:**
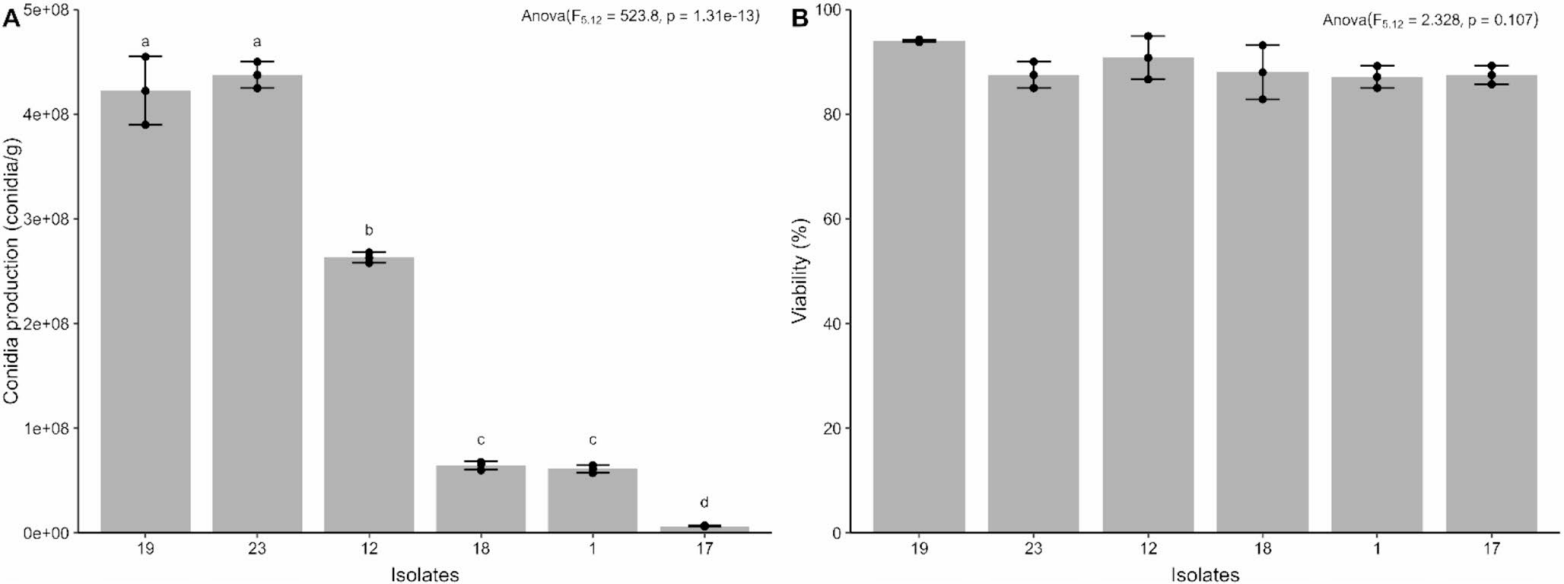
Production (**A**) and viability (**B**) of aerial conidia of entomopathogenic fungi in rice substrate. Isolates with the same letters do not differ significantly according to Duncan’s test (*p* < 0.05)

Significant variation in conidial productivity was observed among the tested isolates (F_5.12_ = 523.8, *p* = 1.31 × 10^− 13^), with values ranging from 6.58 × 10⁶ to 4.37 × 10⁸ conidia/g of inoculated rice. Isolates 23 and 19, both belonging to the species *B. bassiana*, showed the highest conidial production productivities, with 4.37 × 10⁸ and 4.22 × 10⁸ conidia/g, respectively, not differing statistically from each other by Duncan’s test (*p* < 0.05) (Fig. 6A). The viability of conidia produced in rice was also evaluated, with no statistically significant differences detected between the isolates (F_5.12_ = 2.328, *p* = 0.107), with values ranging from 87 to 94% germination (Fig. 6B).

### Production of insect Cuticle-degrading enzymes

Low proteolytic activity was detected in all EPF isolates tested (Table 1). Similarly, chitinolytic activity was considered reduced for most isolates (EI between 1.00 and 1.40), except for isolate 1, which showed no detectable chitinolytic activity, evidenced by the absence of red coloration around its colony when grown in culture medium containing colloidal chitin as an inducer of chitinase production (Table 1; Fig. S1). Regarding lipolytic activity, all EPF isolates showed a positive response, with varying intensity. Isolate 1 showed the highest lipolytic activity, indicated by the intense orange coloration in the Petri dishes (Table 1; Fig. S1).

**Table 1:** Enzymatic activity index of protease, chitinase and lipase of different isolates of entomopathogenic fungi native to the semi-arid region of the state of Sergipe/Brazil

Isolate	Specie	Enzymatic activity					
		Protease	EI	Chitinase	EI	Lipase	EI
Isolate 12	*Beauveria bassiana*	1.11 ± 0.01	L	1.40 ± 0.03	L	1	L
Isolate 17		1.13 ± 0.02	L	1.00 ± 0.00	L	3	G
Isolate 18		1.16 ± 0.03	L	1.00 ± 0.00	L	1	P
Isolate 19		1.11 ± 0.01	L	1.11 ± 0.01	L	2	M
Isolate 23		1.20 ± 0.01	L	1.24 ± 0.09	L	3	G
Isolate 1	*Metarhizium pinghaense*	1.04 ± 0.02	L	0.00 ± 0.00	-	4	E
Mean value	-	1.12 ± 0.05	L	0.95 ± 0.49	L	-	-

EI = Enzymatic activity index. For protease and chitinase: (≤ 1.5 = low activity (L), 1.5–2 = moderate activity (M), 2–3 = good activity (G), > 3 = excellent activity (E)). The lipase enzymatic activity index was evaluated based on a rating scale, ranging from 1 to 4, where: 1 – low activity (L), 2 – moderate activity (M), 3 – good activity (G) and 4 – excellent activity (E)

### Evaluation of the tolerance of isolates of entomopathogenic fungi to different stress

#### Heat stress

**Fig. 7:**
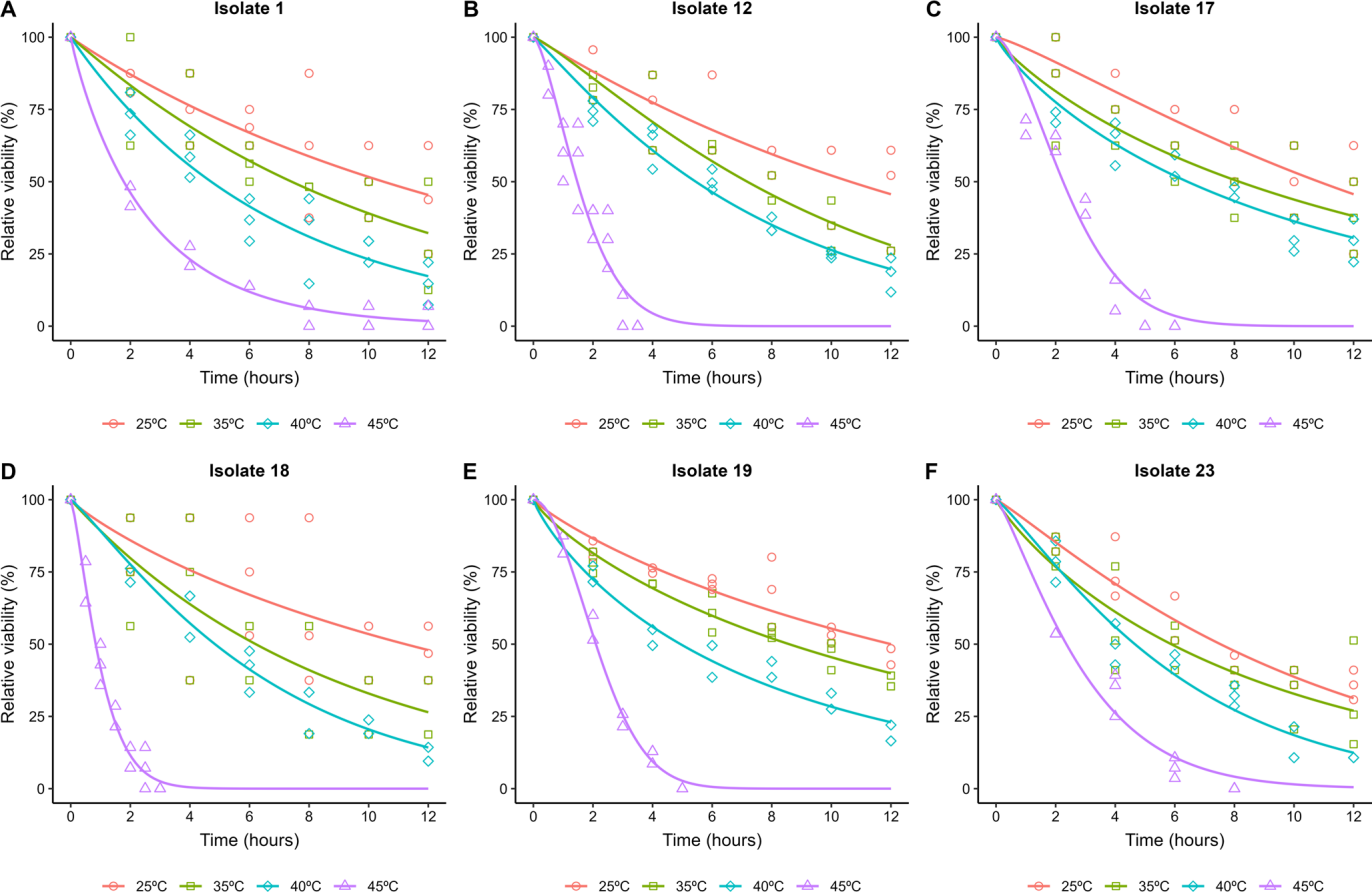
Relative viability of aerial conidia of entomopathogenic fungi exposed to different temperatures from 0 (control) to 12 h. The line indicates the fit of the data to the two-parameter Weibull model

Exposure to temperatures of 25, 35 and 40 °C for 12 h did not completely render the conidia of EPF unviable. Exposure to a temperature of 45 °C promoted a significant reduction in the conidial viability of EPF, with total unfeasibility of the conidia between 3 and 8 h of exposure, for most isolates. Only the isolate 1 (*M. pinghaense*) showed conidial viability after 12 h of exposure to 45 °C (2.3%) (Fig. 7).

**Fig. 8:**
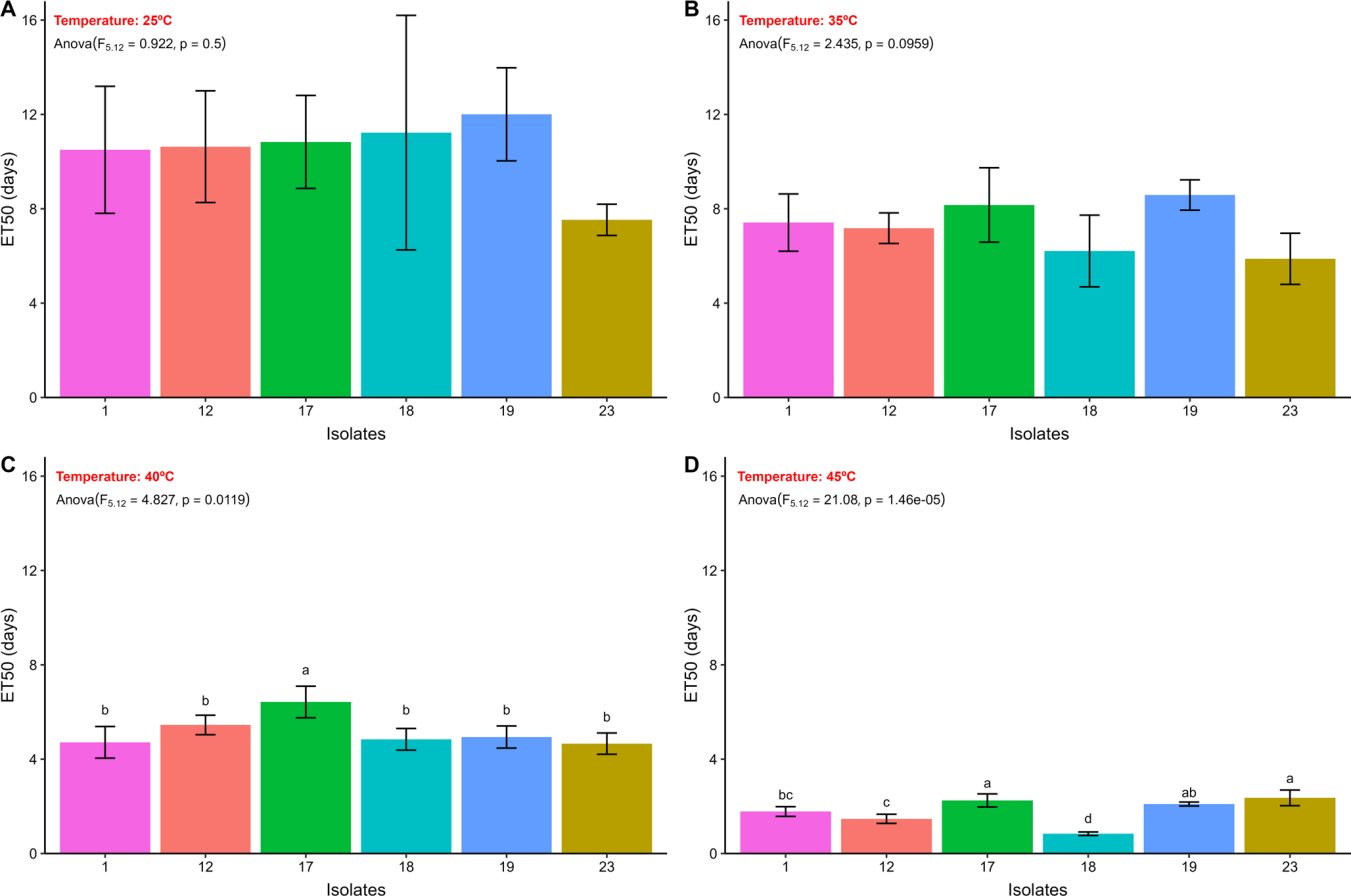
Estimated median effective time to promote a 50% reduction in the relative viability of aerial conidia of entomopathogenic fungi (ET_50_, days) after exposure to different temperatures (**A** = 25 °C, **B** = 35 °C, **C** = 40 °C, **D** = 45 °C), followed by their respective confidence intervals. Similar letters in the results of the different entomopathogenic fungal isolates indicate no significant difference, according to Duncan’s test (*p* < 0.05)

**Fig. 9:**
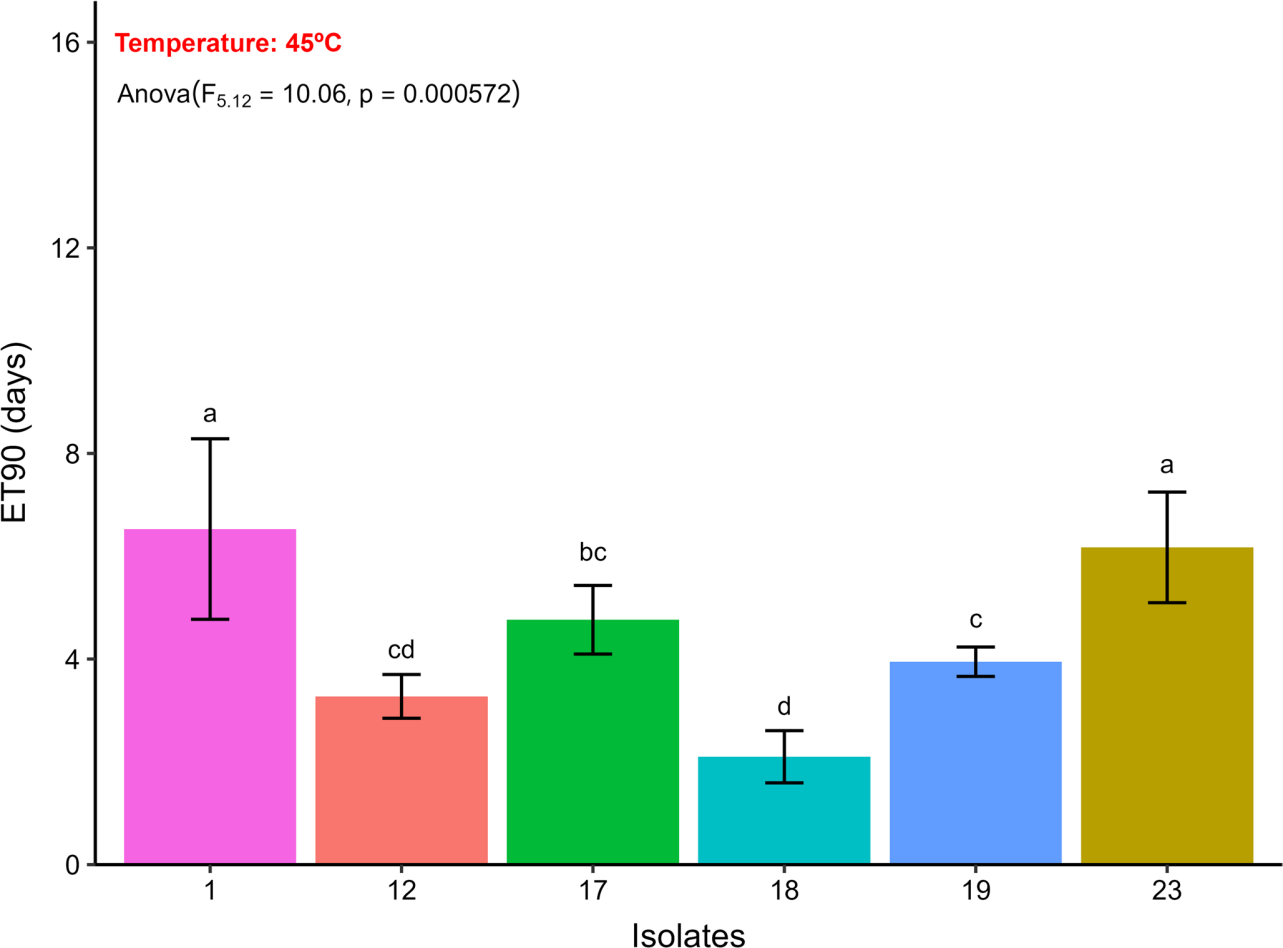
Estimated median effective time to promote a 90% reduction in the relative viability of aerial conidia of entomopathogenic fungi (ET_90_, days) after exposure to 45 °C, followed by their respective confidence intervals. Similar letters in the results of the different entomopathogenic fungal isolates indicate no significant difference, according to Duncan’s test (*p* < 0.05)

Variations in the ET estimate by two-parameter Weibull model were observed depending on the isolate and the temperature tested (Figs. 8 and 9). The isolates presented ET_50_ ranging in: 25 °C: (ET_50_ = 7.53–12 h), 35 °C: (ET_50_ = 5.87–8.16 h), 40 °C: (ET_50_ = 4.65–6.42 h) and 45 °C: (ET_50_ = 0.83–2.35 h). At 25 °C and 35 °C, no significant differences were detected between the ET_50_ values among the different fungal isolates (Fig. 8A and B). However, at 40 °C and 45 °C, significant differences emerged in the estimated ET_50_ values. Specifically, at 40 °C, the isolate 17 displayed the highest estimated ET_50_ value (6.42 h) (Fig. 8C). At 45 °C, isolates 19, 17 and 23 demonstrated the highest estimated ET_50_ values of 2.09, 2.25, and 2.35 h, respectively (Fig. 8D).

All EPF isolates showed a viability reduction of less than 90% at temperatures of 25, 30, and 40 °C after 12 h of exposure (Fig. 9). At 45 °C, variation in the ET_90_ of the EPF isolates was observed (ET_90_ = 2.09–6.52 h). A significant difference in the ET_90_ of the isolates was observed, with isolates 23, and 1 standing out for presenting the highest estimated ET_90_ values, 6.17, and 6.52 days, respectively (Fig. 9).

#### UV-B Radiation stress

**Fig. 10:**
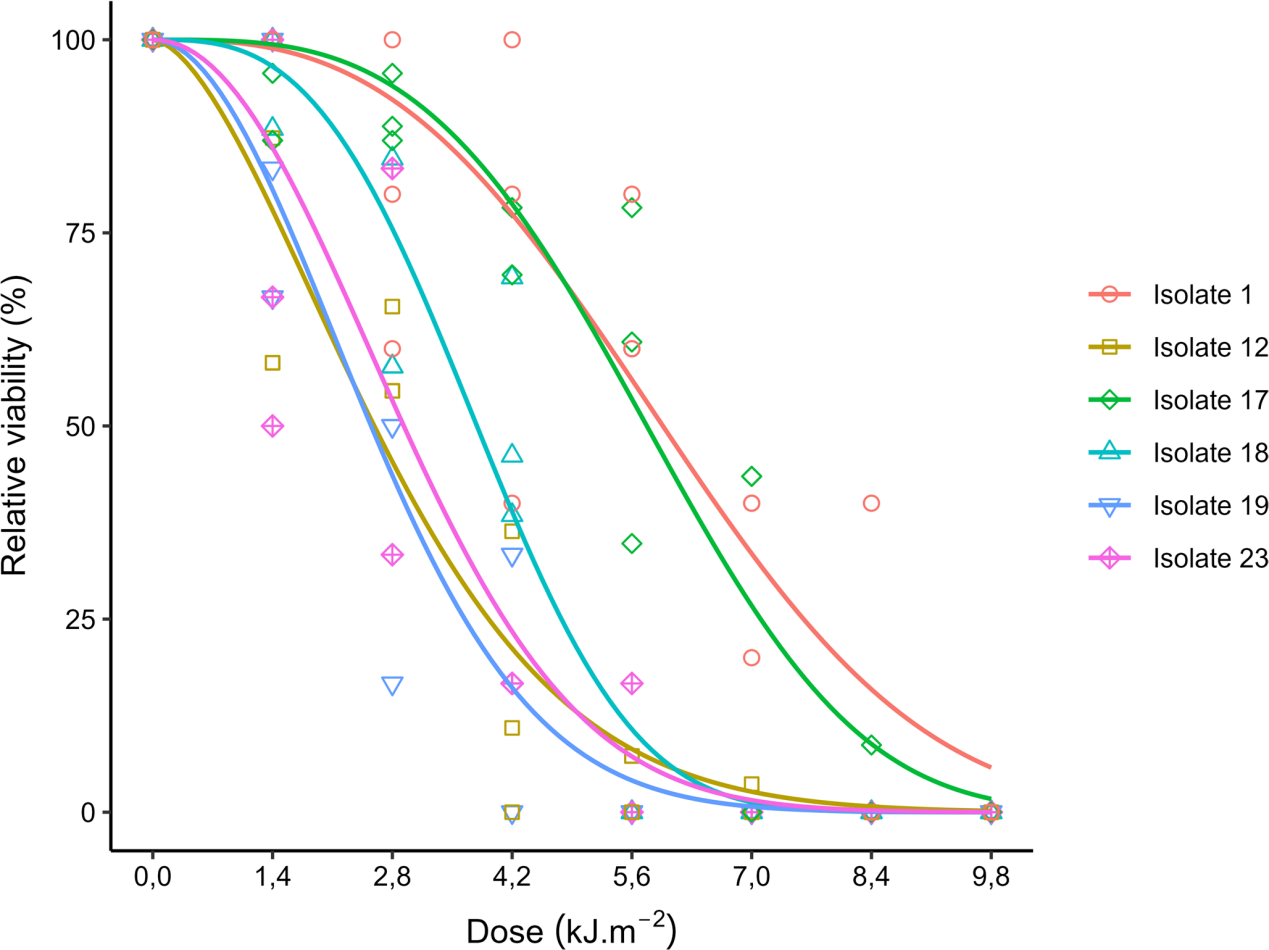
Relative viability of aerial conidia of entomopathogenic fungi exposed to different doses of UV-B radiation in a range from 0 (control) to 9.8 kJ.m^− 2^. The line indicates the fit of the data to the two-parameter Weibull model

**Fig. 11:**
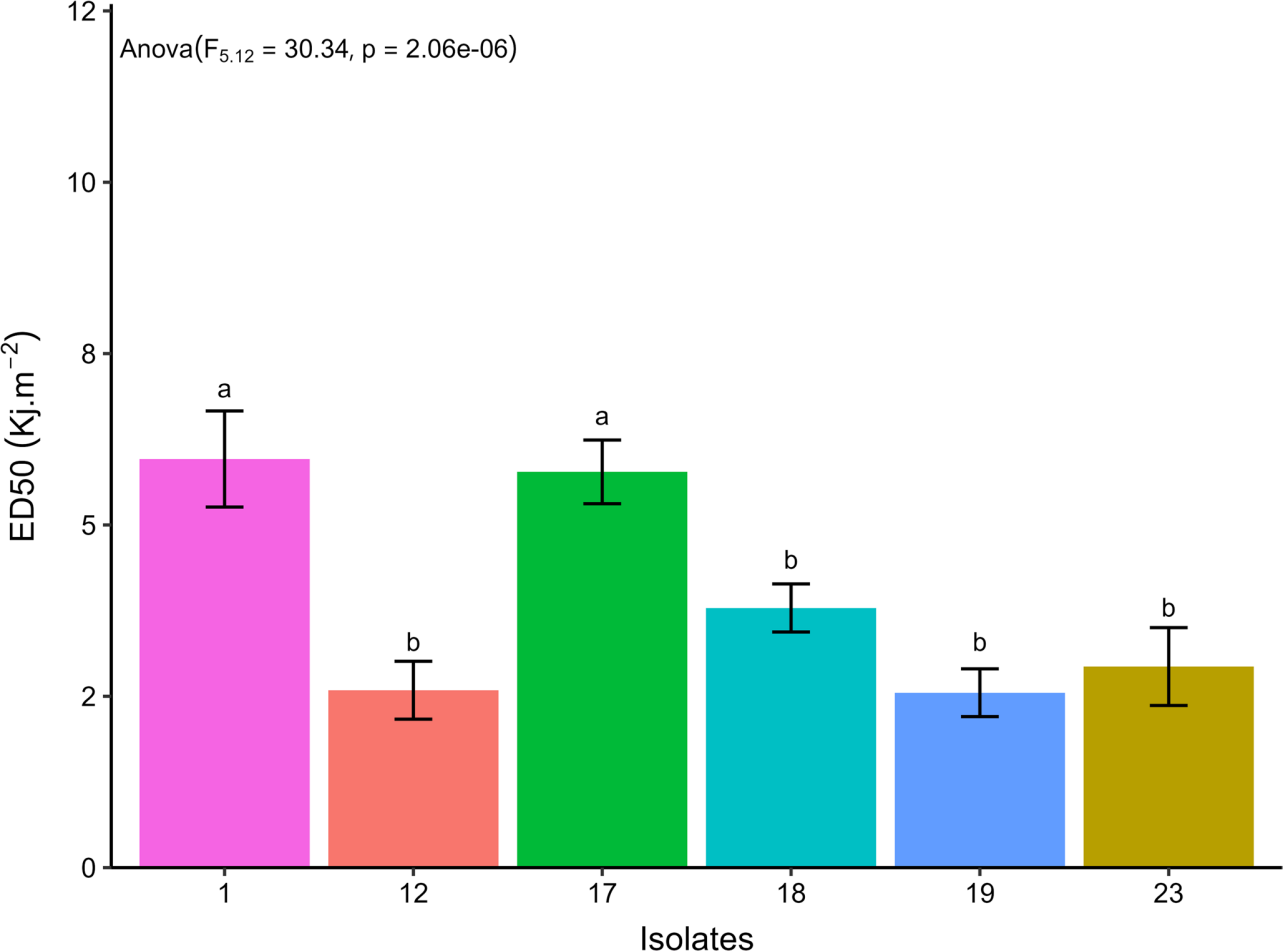
Estimated median effective dose to promote a 50% reduction in the relative viability of aerial conidia of entomopathogenic fungi (ED_50_, days) after exposure to UV-B radiation, followed by their respective confidence intervals. Similar letters in the results of the different entomopathogenic fungal isolates indicate no significant difference, according to Duncan’s test (*p* < 0.05)

**Fig. 12:**
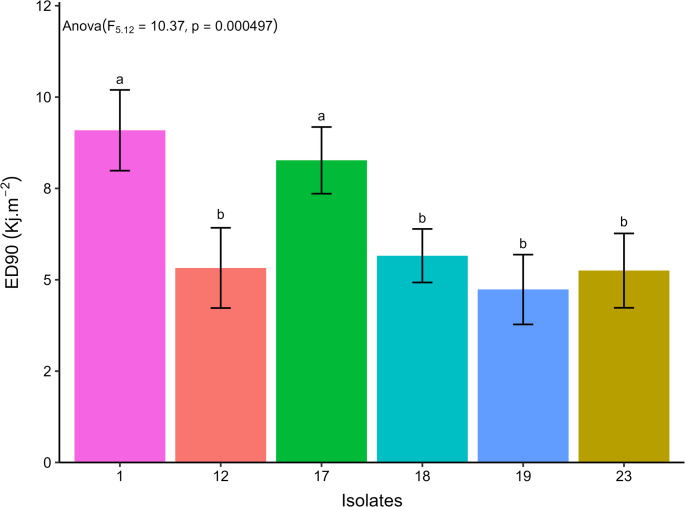
Estimated median effective dose to promote a 90% reduction in the relative viability of aerial conidia of entomopathogenic fungi (ED_90_, days) after exposure to UV-B radiation, followed by their respective confidence intervals. Similar letters in the results of the different entomopathogenic fungal isolates indicate no significant difference, according to Duncan’s test (*p* < 0.05)

The EPF isolates presented ED_50_ ranging from 2.75 to 5.96 kJ.m^− 2^, referring to isolates 19 and 1, respectively (Fig. 10). Isolates 17 and 1 presented the highest estimated values of ED_50_, 5.77 and 5.96 kJ.m^− 2^, and of ED_90_, 8.26 and 9.09 kJ.m^− 2^, respectively, presenting no statistical difference, considering their respective confidence intervals. The other isolates presented estimated ED_90_ ranging from 4.73 to 5.75 kJ.m^− 2^ (Figs. 11 and 12).

### Fungal growth with oxidative stress, osmotic stress and cell wall stress inducers

**Fig. 13:**
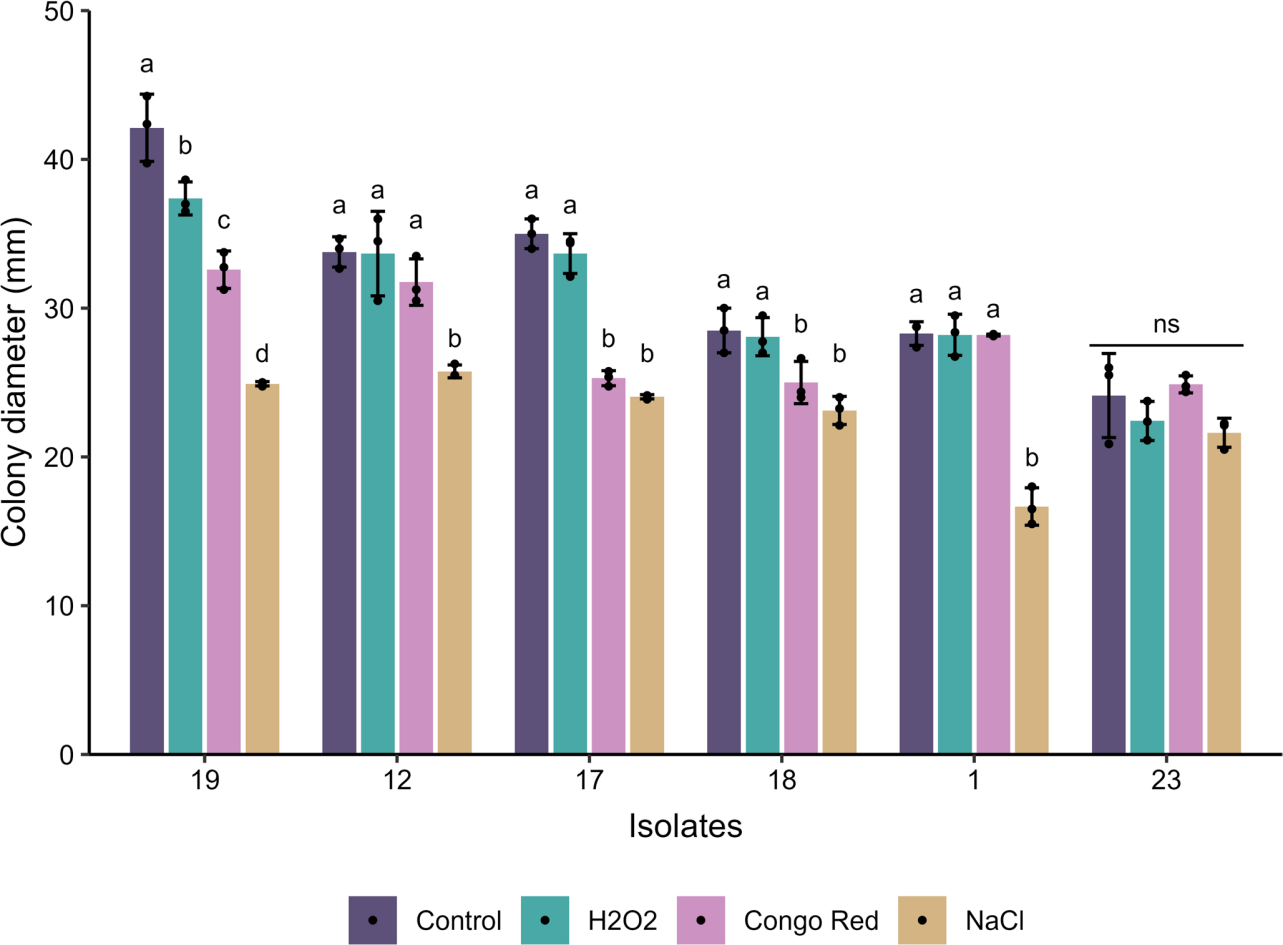
Average diameter of entomopathogenic fungal colonies after exposure to different stress inducers: Hydrogen peroxide (H₂O₂) – oxidative stress, Sodium chloride (NaCl) – osmotic stress and Congo red - stress against cell wall. Bars (± standard deviation) with similar letters do not show significant difference, according to Duncan’s test (*p* < 0.05). ns = not significant

Exposure to inducers of oxidative stress, osmotic stress and stress against the cell wall promoted significant differences in the mycelial growth of most EPF isolates tested (Isolate 1: F_3.8_ = 97.144, *p* < 0.001; Isolate 12: F_3.8_ = 14.552, *p* = 0.001; Isolate 17: F_3.8_ = 124.231, *p* < 0.001; Isolate 18: F_3.8_ = 11.603, *p* = 0.003; Isolate 19: F_3.8_ = 81.365, *p* < 0.001). Only isolate 23 did not show statistically significant changes in mycelial growth when exposed to the evaluated stressors (F_3.8_ = 2.454, *p* = 0.138) (Fig. 13 and S2).

High osmolarity, induced by the addition of NaCl to the medium, was the most limiting factor for mycelial growth for isolates 1, 12, 18, and 19. Osmotic and cell wall stress primarily affected the development of isolate 17. Isolates 1, 12, 17, and 18 demonstrated tolerance to oxidative stress, with relatively preserved mycelial growth. It is also worth noting that isolates 1 and 12 did not show significant growth inhibition upon exposure to Congo red dye (Fig. 13).

## Discussion

Soil is a known reservoir of microorganisms, with about 10^5^−10^6^ fungal cells/g of soil, among which are spores of several species of EPF (Gentry et al. 2021) and has already been used for prospection of EPF in various parts of the world (Fernández-Bravo et al. 2021; Alfiky 2022; Domingues et al. 2022; Yakubu et al. 2022).

Studies have also shown the presence of microbial diversity in the soil of the Brazilian semi-arid region, but without focusing on evaluating the presence of EPF (Oliveira et al. 2013; Pacchioni et al. 2014; Lacerda-Junior et al. 2019; Pinheiro et al. 2024). Our study revealed the presence of EPF in 58.3% of soil samples from the semi-arid region of the state of Sergipe/Brazil, with samples from three cities (Aquidabã, Monte Alegre and Poço Redondo) showing an absence of EPF, using the insect bait isolation method. The low recovery of EPF in soil samples has already been reported by Yakubu et al. (2022), who observed the occurrence of EPF in 41% of soil samples collected in Nigeria, using the insect-bait isolation method. Surveys of EPF in soils from Ethiopia and Saudi Arabia also showed low densities of these microorganisms, 21.4% and 27.3%, respectively (Mekonnem et al. 2024; Sutanto et al. 2024). The absence of EPF observed in some soil samples from our study may be caused by the low density of EPF in the samples, the heterogeneous distribution of fungi in the soil of the region, the biotic and abiotic conditions of the soil (moisture, organic matter content), or the influence of the method used to isolate microorganisms (Hallouti et al. 2020; Tedersoo et al. 2020; Afandhi et al. 2022; Côrrea et al. 2022).

The isolates obtained had their pathogenic potential confirmed through a pathogenicity assessment. The confirmation of the pathogenicity of the isolates is part of the steps proposed by Koch’s Postulate for the determination of a causal agent of diseases. The use of this method allows for greater accuracy in the effectiveness of the isolation of EPF, since some filamentous fungi widely present in the soil, such as *Fusarium* spp., can eventually colonize insects due to their saprophytic action (Alfiky 2022; Karim et al. 2016). Despite using *T. molitor* larvae as bait for isolating EPF from soil, in our study, only 2 of the 14 fungal isolates prospected showed high pathogenicity (> 80%) against *T. molitor* larvae. The occurrence of variation in the pathogenicity of EPF isolates when applied to the same insect species from which they were isolated is recurrent (Kim et al. 2018; Chang et al. 2021; Eski and Gezgin 2022) and may be associated with differences in concentration between the fungus in the soil and in the bioassay, greater susceptibility to infection in the insect-bait method, or selectivity of the isolated fungi.

Despite this, 5 isolates showed high pathogenicity (> 80%) and 1 isolate showed moderate pathogenicity (60–80%) in the preliminary pathogenicity bioassay of EPF on *P. xylostella*, according to the scale described by Chang et al. (2021). The difference observed between the pathogenicity of the fungal isolates tested on *T. molitor* and *P. xylostella* may indicate that some isolates present specificity of action against *P. xylostella* and not against *T. molitor*, being fungi with high insecticidal potential, despite these characteristics. Therefore, EPF isolates 1, 12, 17, 18, 19 and 23 were selected as the most promising for further analysis.

EPF isolates prospected in our study were identified as *B. bassiana* and *M. pinghaense* species. Genus *Beauveria* and *Metarhizium* are commonly collected in soil samples or by colonizing insects of various orders in different regions of the world, with *B. bassiana* being one of the most prospected and used species in the biological control of pest insects (Irsad et al. 2023). Among the species of the genus *Metarhizium*, *Metarhizium anisopliae* is the species most used in biological pest control. However, there are indications that all species of clade 1 of the genus *Metarhizium*, formed by *M. majus*, *M. guizhouense*, *M. pinghaense*, *M. anisopliae*, *M. robertsii* and *M. brunneum*, are found in soils, mainly in samples from pastures and arable land, and these places may be natural reservoirs of these species (Fernàndez-Bravo et al. 2021).

The selected fungi were subjected to a further stage of insecticidal evaluation with the aim of confirming their pathogenicity and assessing their virulence (LT_50_) against *P. xylostella* (the target pest of the study). All fungal isolates showed a high rate of pathogenicity against the target insect, confirming their pathogenic potential; however, there were differences in their virulence, based on the LT_50_ estimate. The high genetic variability of EPF isolates promotes intra- and interspecific variations in their virulence against *P. xylostella* (Shehzad et al. 2021; Abarma et al. 2024). Therefore, the variation in LT_50_ observed in our isolates may.be related to the existence of phenotypic and genomic variation in the evaluated isolates, such as variation in the expression of genes related to the production of toxins and enzymes involved in the degradation of pest tissues or genes for stress resistance (Huang et al. 2022; Parker et al. 2023).

The pathogenicity and virulence of EPF are associated with their potential to produce metabolites that facilitate their adhesion, penetration, or infestation of host tissues. Among these, hydrolytic enzymes that act in fungal penetration (proteases, lipases, and chitinases) are the most frequently reported to contribute to the virulence of these microorganisms (Mannino et al., 2019; Pelizza et al. 2017; Gotti et al. 2023; Iwanicki et al. 2023). Therefore, isolates of EPF were also evaluated for their potential to produce these enzymes in assays on solid culture media with qualitative assessment inducers, to assess whether there was an association between the pathogenicity and virulence of the fungi and their enzymatic production. However, in our study, no correlation was observed between the production of extracellular proteases, chitinases, and lipases and the pathogenicity of the isolates tested in *P. xylostella*. These results indicate that, despite being important for the infection of EPF in their hosts, the hydrolytic enzymes required in the penetration process of EPF are not directly related to the virulence of the isolates obtained in our study. Reports in scientific literature present divergent results on this type of comparison. Some studies report the existence of a positive correlation between virulence and the production of protease, lipase, and chitinase by fungi (Dhawan and Joshi 2017); however, some studies infer the absence of a clear correlation between the virulence of EPF and the protease production (Mascarin et al. 2013), or even the correlation of protease and chitinase with fungal virulence and the absence of correlation with lipase production (Shahriari et al. 2021). This occurs due to the genetic variability of the isolates used in the studies and the different insect species used as study models. However, our study provides a qualitative assessment of the production of these enzymes by the EPF isolates studied. Therefore, future research on metabolites related to the pathogenicity and virulence of the isolates obtained in our study is necessary for a better understanding of their pathogen-host interaction.

For the use of EPF in the control of agricultural pests, the large-scale production of their conidia is necessary. Therefore, the mass production stage is important to enable the use of this biological agent in crops. Based on this, the evaluation of mass conidia production was added as a step in the selection of the most promising EPF isolates to be subjected to large-scale conidia production. In our study, observed variation in the aerial conidial production of entomopathogenic in solid-state fermentation with rice substrate, most used grain for this purpose. Furthermore, some of our *B. bassiana* isolates showed greater potential for aerial conidial production compared to data from other studies reported in the literature, in which conidial yield ranged from 2 × 10^6^ and 1 × 10^8^ conidia/g (Kaur and Joshi 2014; Paiva-Guimarães et al. 2019; Rai et al. 2021). No records of *M. pinghaense* production using rice as growth substrate were found. In general, the conidia produced in the rice substrate has a high germination rate, often above 80%.

Different types of cellular stress (heat stress, UV-B radiation stress, oxidative stress, osmotic stress, and cell wall stress) occurring during storage or field application affect the viability and cellular development of EPF. Therefore, it has been necessary to understand how these types of stress impact the vigor of different EPF species (Bernardo et al. 2020; Wu et al. 2020; Couceiro et al. 2021; Gava et al. 2022; Licona-Juárez et al. 2023; Rangel et al. 2023). EPF isolated in our study exhibits intraspecific and interspecific variations in stress tolerance. For example, isolates of *B. bassiana* showed contrasting responses to thermal exposure: while some were completely unfeasible after exposure to 40 °C (Gava et al. 2022), others maintained 50% germination after exposure to 45 °C for 40 min (Bernardo et al. 2020). An analogous situation was observed with *M. anisopliae*, whose conidia of certain isolates were totally unfeasible at 40 °C, while another isolate, of different origin, presented ET_50_ of 125.59 min (2.09 h) at 45 °C. Notably, one isolate of *M. acridum* exhibited high thermal resistance, with ET_50_ estimated at 7.25 h at 45 °C (Bernardo et al. 2020).

In our study, it was observed that the isolates of EPF tested showed tolerance to exposure to 25, 35 and 40 °C, for more than 12 h, and ET_90_ between 2.09 and 6.52 h for exposure to 45 °C. These results may be related to intrinsic characteristics of these isolates, related to their isolation origin (possibly related to the edaphoclimatic and ecological conditions of their geographic origin). In addition, the thermal tolerance of EPF may be associated with increased pyruvate production, which reduces the effect of ROS (reactive oxygen species) on cells, and the positive regulation of protein production with action to protect against heat stress (Wu et al. 2019; Xie et al. 2019; Zhang et al. 2024).

Tolerance to UV radiation in EPF varies depending on the fungus evaluated. Isolates of *B. bassiana*, *M. robertsii*, M. *anisopliae*, and *M. acridum* showed variation in the effective dose to inactivate 50% (ED_50_) of their aerial conidia (3.96–12.26 kJ.m^− 2^), with one isolate of *M. anisopliae* being completely inactivated after 10 h (Bernardo et al. 2020). However, Couceiro et al. (2021) observed that most *Metarhizium* isolates in their study were inactivated after 8 h of exposure to UV radiation (19.04 kJ.m^− 2^), although one isolate (*M. robertsii*) showed 50% germination when subjected to similar conditions. The expression of genes to produce enzymes with DNA repair action, such as photolyase enzymes, observed in *M. pinghaense*, or laccase production, related to the production of pigment by fungi, are factors associated with the tolerance of EPF against UV-B radiation (Fang et al. 2010; Lima et al. 2023). These physiological mechanisms of reduced susceptibility to UV-B radiation may be responsible for the greater tolerance of isolates 1 and 17, when compared to the other isolates tested in this study.

Oxidative stress in EPF occurs when the microorganism’s antioxidant enzymes fail to suppress the action of reactive oxygen species (ROS) on cells. ROS in EPF results from environmental fluctuations (temperature, radiation), interactions with the host insect’s immune system during infection, and exposure to toxic compounds in the environment. Excess ROS in cells promotes damage to cellular components (lipids, proteins, and DNA) and impairs their functions (Martins et al. 2013; Huarte-Bonnet et al. 2019, Litwin et al. 2021). In the present study, the isolate 19 (*B. bassiana*) was the only one that presented significant sensitivity to oxidative stress induced by H₂O₂, showing a lower physiological capacity to neutralize ROS, when compared to the other isolates.

Exposure to high concentrations of osmolytes induces the development of osmotic stress in cells, which causes modifications in the cytosol and can increase the cell’s energy requirement for the development of its functions, which can cause dehydration and even cell death (Blomberg and Adler 1992; Saito and Posas 2012). Some fungi are tolerant of osmotic stress, and this characteristic may be related to their ability to perform physiological modifications to adjust their internal water activity or because they are able to reduce the permeability of their plasma membrane (Turk et al. 2003; Rangel et al. 2015). In a previous study, it was observed that *B. bassiana* and *Metarhizium* spp. have a high susceptibility to osmotic stress (Araújo et al. 2019). In our study, it was observed that osmotic stress, induced by the presence of NaCl, was the most harmful to the development of EPF, when compared to the evaluation of oxidative stress and against the cell wall, promoting a significant reduction in mycelial growth (Figs. 13 and S2). The isolate 23 (*B. bassiana*) was the only entomopathogenic fungus tested that did not show a significant reduction in its mycelial growth in the presence of NaCl, being the most tolerant of osmotic stress.

Tolerance to stress against the cell wall is commonly accomplished with the use of Congo red dye, that do not penetrate the cells but bind to chitin and proteins present in the cell wall of fungi., destabilizing them and causing morphological changes in their cell wall (Levin 2005; Ram and Klis 2006). The isolate 1 (*M. pinghaense*) and isolates 12 and 23 (*B. bassiana*) showed high tolerance to the action of the Congo red dye, indicating that these isolates have a more resistant cell wall than the other fungi tested. The literature points out that entomopathogenic species such as *B. bassiana* and *Metarhizium* spp. are generally less tolerant to the action of Congo red compared to saprophytic fungi such as *Fusarium* spp. and *Trichoderma atroviride* (Lima et al. 2021). However, the presence of protective mechanisms, such as calcineurin activation, may favor the maintenance of cell wall integrity in certain isolates (Huang et al. 2015). These results suggest important phenotypic variations among isolates in response to different types of stress, reflecting distinct physiological mechanisms of adaptation and ecological potential, with direct implications for the selection of strains with greater robustness for use in environments with adverse conditions.

The combined evaluation of data from characterization assays of EPF suggests that isolate 1 is the best candidate for use in pest control, as it presented the best results in most of the evaluated criteria, such as high pathogenicity and virulence against *P. xylostella*, high lipase production, greater tolerance to UV radiation and thermal stress, and tolerance to oxidative stress and cell wall stress (Table 2). An important observation regarding this isolate is its high pathogenicity and virulence, even with low protease production and absence of chitinase secretion. The pathogenicity stage of EPF is biochemically mediated by several enzymes and secondary metabolites, with protease, lipase, and chitinase considered the main enzymes involved in the penetration of the fungus into the host (Vidhate et al. 2022). In this sense, among the enzymes mediating infection, isolate 1 show only high secretion of lipase. The high secretion of this enzyme may be associated with a compensatory mechanism of the fungus to enable infection in the host even without the effective participation of the other enzymes. Furthermore, other metabolites not evaluated, such as destruxins and trehalose-degrading enzymes, may also mediate the infection caused by this isolate in the insect host, influencing its pathogenicity and virulence (Karthi et al. 2024).

**Table 2:** Summarized data from characterization assays of entomopathogenic fungal isolates from the Brazilian semi-arid region

Isolates	Pathogenicity	Virulence (LT_50_ - days)	Conidia production (conidia/g)	Enzyme production			Stress tolerance				
				Protease	Chitinase	Lipase	Heat stress (ET_90_ – 45 °C)	UV stress (ED_90_)	Oxidative stress	Osmotic stress	Cell wall stress
Isolate 1	97.34 ± 1.77% a	3	6.13 × 10^7^ ± 0.37 c	L	-	E	6.52 (4.77–8.28) a	9.09 (7.98–10.19) a	T	NT	T
Isolate 12	91.29 ± 4.37% a	5	2.63 × 10^8^ ± 0.05 b	L	L	L	3.27 (2.84–3.69) cd	5.32 (4.22–6.42) b	T	NT	T
Isolate 17	81.51 ± 8.64% b	4	6.58 × 10^6^ ± 0.70 d	L	L	G	4.76 (4.03–5.43) bc	8.26 (7.35–9.18) a	T	NT	NT
Isolate 18	93.61 ± 3.17% a	4	6.44 × 10^7^ ± 0.39 c	L	L	P	2.09 (1.59–2.60) d	5.65 (4.92–6.39) b	T	NT	NT
Isolate 19	96.60 ± 2.37% a	3	4.23 × 10^8^ ± 0.32 a	L	L	M	3.94 (3.66–4.23) c	4.73 (3.77–6.42) b	NT	NT	NT
Isolate 23	82.22 ± 7.44% b	4	4.38 × 10^8^ ± 0.12 a	L	L	G	6.17 (5.09–7.24) a	5.25 (4.23–6.26) b	T	T	T

LT_50_ = Time required for entomopathogenic fungi to cause death in 50% of treated insects

*EI * Enzymatic activity index, *L* Low activity, *M* Moderate activity, *G* Good activity, *E* Excellent activity. Stress tolerance: *T* Tolerant, *NT* Not tolerant

ET_90_ = Effective temperature required for the inactivation of 90% of the treated conidia. ED_90_ = Effective UV-B dose required for the inactivation of 90% of the treated conidia

## Conclusions

The soil of semi-arid region of Sergipe (Brazil) enabled the isolation of *B. bassiana* and *M. pinghaense* isolates with high pathogenicity and virulence against insect-pests. Relevant intra- and interspecific differences were observed regarding virulence, conidia production, and tolerance to thermal, oxidative, osmotic stresses, UV-B radiation, and cell wall integrity. These variations indicate the existence of metabolic and physiological differences among the isolates, reflecting their adaptability to different environmental conditions. In view of the results obtained, it is concluded that the EPF from the soils of the semi-arid region of Sergipe present promising attributes for their use in the control of agricultural insect pests, also highlighting the importance of selection and characterization tests for the prospecting of EPF isolates for use in the biological control of pests.

## Supplementary Information

Below is the link to the electronic supplementary material.

Supplementary Material 1 (DOCX 1.80 MB)

## Data Availability

The sequencing data of the entomopathogenic fungal isolates prospected in our study are available in a public database (GenBank) under accession numbers: *ITS* = PP506014 - PP506018, *TEF-1* = PP590613 and *B-TUB* = PP598662 – PP598667.
