# Supplementary material for: Bioprospection of entomopathogenic fungi natives of Brazilian Semi-arid with potential for biological control of Insect-pests
Source: World J Microbiol Biotechnol. 2026 Apr 18;42(5):217. doi: 10.1007/s11274-026-04946-6 (PMC13090199; doi:10.1007/s11274-026-04946-6)
Supplement: Supplementary file 1 — Supplementary Material 1 (DOCX 1.80 MB) [file 11274_2026_4946_MOESM1_ESM.docx]

**Supplementary material**

**Bioprospection of entomopathogenic fungi natives of Brazilian semi-arid with potential for biological control of insect-pests**

Tárcio Souza Santos^1^, Jackson Freitas de Almeida Santos^2^, Emilly Lourdes Tavares Santos^2^, Thomaz Soares Santos^1^, Josefa Lívia Silva Leite^1^, Arie Fitzgerald Blank^1^, Marcelo da Costa Mendonça^2,3^.

Table S1: Soil collection locations, vegetation type and coordinates of the different sampling points in the semi-arid region of the state of Sergipe, Brazil.

| **Sample number** | **Collection locations / City** | **Vegetation type** | **Geographical coordinates** |
| --- | --- | --- | --- |
| 1 | Frei Paulo | Crop/Palm | S -10°30'00.0'' / W -37°39'16.8'' |
| 2 | Frei Paulo | Native vegetation | S -10°30'00.0'' / W -37°39'21.0'' |
| 3 | Frei Paulo | Crop / Maize | S -10°28'42.4'' / W -37°39'31.2'' |
| 4 | Carira | Pasture | S -10°21'04.5'' / W -37°40'45.0'' |
| 5 | Carira | Crop / Maize | S -10°17'37.9'' / W -37°42'06.2'' |
| 6 | Carira | Crop / Maize | S -10°21'54.0'' / W -37°42'23.8'' |
| 7 | Pinhão | Crop / Maize | S -10°33'44.3'' / W -37°42'14.9'' |
| 8 | Pinhão | Native vegetation | S -10°37'10.0'' / W -37°44'57.7'' |
| 9 | Simão Dias | Crop / Maize | S -10°40'35.5'' / W -37°45'17.9'' |
| 10 | Simão Dias | Crop/Banana | S -10°41'47.4'' / W -37°46'25.5'' |
| 11 | Simão Dias | Native vegetation | S -10°43'49.1'' / W -37°46'47.5'' |
| 12 | Canindé de São Francisco | Native vegetation | S -09°40'28.7'' / W -37°45'28.4'' |
| 13 | Poço Redondo | Crop / Okra | S -09°41'24,0'' / W -37°44'30.3'' |
| 14 | Poço Redondo | Crop/Banana | S -09°41'22.3'' / W -37°44'30.9'' |
| 15 | Poço Redondo | Crop/Palm | S -09°50'02.1'' / W -37°40'18.4'' |
| 16 | Monte Alegre | Crop/Palm | S -10°03'22.8'' / W -37°33'15.5'' |
| 17 | Nossa Senhora da Glória | Native vegetation | S -10°12'29.3'' / W -37°31'33.3'' |
| 18 | Nossa Senhora da Glória | Native vegetation | S -10°11'55,3'' / W -37°33'00,6'' |
| 19 | Nossa Senhora da Glória | Native vegetation | S -10°11'13.5'' / W -37°34'02.5'' |
| 20 | Nossa Senhora da Glória | Crop / Maize | S -10°12'29.7'' / W -37°31'33.2'' |
| 21 | Canindé de São Francisco | Crop / Maize | S -09°41'56.9'' / W -37°53'12.0'' |
| 22 | Canindé de São Francisco | Native vegetation | S -09°41'47.5'' / W -37°50'37.7'' |
| 23 | Gararu | Crop/Palm | S -09°56'00.7'' / W -37°13'47.0'' |
| 24 | Aquidabã | Pasture | S -10°10'08.2'' / W -37°08'59.5'' |

Table S2: Best alignments obtained by Blastn (NCBI) for identification of *Beauveria* (*ITS* region and *B-TUB* gene) and *Metarhizium* (*TEF-1* and *B-TUB* gene) isolates.

| Description | Scientific name | Max score | Total score | Query cover | E value | Per. Ident. | Acc. Len | Accession | |
| --- | --- | --- | --- | --- | --- | --- | --- | --- | --- |
| ***Beauveria* identification – *ITS* region** | | | | | | | | | |
| **Isolate 12** | | | | | | | | | |
| *Beauveria bassiana* strain UFRA_Bb02 small subunit ribosomal RNA gene, partial sequence | *Beauveria bassiana* | 1014 | 1014 | 100% | 0.0 | 100.00% | 562 | MN947543.1 | |
| *Beauveria bassiana* isolate SASRI C2 18S ribosomal RNA gene, partial sequence | *Beauveria bassiana* | 1014 | 1014 | 100% | 0.0 | 100.00% | 594 | JX110371.1 | |
| *Cordyceps bassiana* internal transcribed spacer 1, partial sequence | *Beauveria bassiana* | 1014 | 1014 | 100% | 0.0 | 100.00% | 579 | EF672309.1 | |
| *Beauveria bassiana* isolate 1.15 small subunit ribosomal RNA gene, partial sequence | *Beauveria bassiana* | 1014 | 1014 | 100% | 0.0 | 100.00% | 551 | MH233337.1 | |
| *Beauveria bassiana* strain Ya1 small subunit ribosomal RNA gene, partial sequence | *Beauveria bassiana* | 1014 | 1014 | 100% | 0.0 | 100.00% | 593 | MK418845.1 | |
| *Beauveria bassiana* isolate strain RGM 565 small subunit ribosomal RNA gene, partial sequence | *Beauveria bassiana* | 1014 | 1014 | 100% | 0.0 | 100.00% | 563 | MH231198.1 | |
| *Beauveria bassiana* isolate B13 small subunit ribosomal RNA gene, partial sequence | *Beauveria bassiana* | 1014 | 1014 | 100% | 0.0 | 100.00% | 585 | MH922794.1 | |
| *Beauveria bassiana* genomic DNA containing ITS1, 5.8S rRNA and ITS2, strain Mas14, isolate A | *Beauveria bassiana* | 1014 | 1014 | 100% | 0.0 | 100.00% | 579 | LN823583.1 | |
| *Beauveria bassiana* strain B-Bug small subunit ribosomal RNA gene, partial sequence | *Beauveria bassiana* | 1014 | 1014 | 100% | 0.0 | 100.00% | 638 | MK862359.1 | |
| *Beauveria bassiana* isolate 1.24 small subunit ribosomal RNA gene, partial sequence | *Beauveria bassiana* | 1014 | 1014 | 100% | 0.0 | 100.00% | 559 | MH233346.1 | |
| **Isolate 17** | | | | | | | | | |
| Uncultured fungus clone OTU_19 18S ribosomal RNA gene, partial sequence | Uncultured fungus | 1014 | 1014 | 100% | 0.0 | 100.00% | 594 | KM032322.1 | |
| *Cordyceps sp*. 97005 gene for 18S rRNA, ITS1, 5.8S rRNA, ITS2, 26S rRNA | *Cordyceps sp* | 1014 | 1014 | 100% | 0.0 | 100.00% | 1658 | AB044636.1 | |
| *Beauveria bassiana* isolate 4508 18S ribosomal RNA gene, partial sequence | *Beauveria bassiana* | 1014 | 1014 | 100% | 0.0 | 100.00% | 570 | KX901307.1 | |
| *Beauveria bassiana* isolate 1558 18S ribosomal RNA gene, partial sequence | *Beauveria bassiana* | 1014 | 1014 | 100% | 0.0 | 100.00% | 571 | AY531984.1 | |
| *Beauveria bassiana* isolate J05 small subunit ribosomal RNA gene, partial sequence | *Beauveria bassiana* | 1014 | 1014 | 100% | 0.0 | 100.00% | 598 | PQ535529.1 | |
| *Beauveria bassiana* BCRC:FU31669 genes for 18S rRNA, ITS1, 5.8S rRNA, ITS2 | *Beauveria bassiana* | 1014 | 1014 | 100% | 0.0 | 100.00% | 713 | LC768985.1 | |
| *Beauveria bassiana* isolate J28 small subunit ribosomal RNA gene, partial sequence | *Beauveria bassiana* | 1014 | 1014 | 100% | 0.0 | 100.00% | 586 | PQ535533.1 | |
| *Beauveria bassiana* isolate pentatomidae small subunit ribosomal RNA gene, partial sequence | *Beauveria bassiana* | 1014 | 1014 | 100% | 0.0 | 100.00% | 584 | PQ534327.1 | |
| *Beauveria bassiana* isolate BRM068905 small subunit ribosomal RNA gene, partial sequence | *Beauveria bassiana* | 1014 | 1014 | 100% | 0.0 | 100.00% | 549 | PP506015.1 | |
| *Beauveria bassiana* isolate 4511 18S ribosomal RNA gene, partial sequence | *Beauveria bassiana* | 1014 | 1014 | 100% | 0.0 | 100.00% | 570 | KX901310.1 | |
|  | | | | | | | | | |
| **Isolate 18** | | | | | | | | | |
| *Beauveria bassiana* strain UFRA_Bb02 small subunit ribosomal RNA gene, partial sequen | *Beauveria bassiana* | 1014 | 1014 | 100% | 0.0 | 100.00% | 562 | MN947543.1 | |
| *Beauveria bassiana* isolate SASRI C2 18S ribosomal RNA gene, partial sequence | *Beauveria bassiana* | 1014 | 1014 | 100% | 0.0 | 100.00% | 594 | JX110371.1 | |
| *Cordyceps bassiana* internal transcribed spacer 1, partial sequence | *Beauveria bassiana* | 1014 | 1014 | 100% | 0.0 | 100.00% | 579 | EF672309.1 | |
| *Beauveria bassiana* isolate 1.15 small subunit ribosomal RNA gene, partial sequence | *Beauveria bassiana* | 1014 | 1014 | 100% | 0.0 | 100.00% | 551 | MH233337.1 | |
| *Beauveria bassiana* strain Ya1 small subunit ribosomal RNA gene, partial sequence | *Beauveria bassiana* | 1014 | 1014 | 100% | 0.0 | 100.00% | 593 | MK418845.1 | |
| *Beauveria bassiana* isolate strain RGM 565 small subunit ribosomal RNA gene, partial sequence | *Beauveria bassiana* | 1014 | 1014 | 100% | 0.0 | 100.00% | 563 | MH231198.1 | |
| *Beauveria bassiana* isolate B13 small subunit ribosomal RNA gene, partial sequence | *Beauveria bassiana* | 1014 | 1014 | 100% | 0.0 | 100.00% | 585 | MH922794.1 | |
| *Beauveria bassiana* genomic DNA containing ITS1, 5.8S rRNA and ITS2, strain Mas14 | *Beauveria bassiana* | 1014 | 1014 | 100% | 0.0 | 100.00% | 579 | LN823583.1 | |
| *Beauveria bassiana* strain B-Bug small subunit ribosomal RNA gene, partial sequence | *Beauveria bassiana* | 1014 | 1014 | 100% | 0.0 | 100.00% | 638 | MK862359.1 | |
| *Beauveria bassiana* isolate 1.24 small subunit ribosomal RNA gene, partial sequence | *Beauveria bassiana* | 1014 | 1014 | 100% | 0.0 | 100.00% | 559 | MH233346.1 | |
|  | | | | | | | | | |
| **Isolate 19** | | | | | | | | | |
| *Beauveria bassiana* strain UFRA_Bb02 small subunit ribosomal RNA gene, partial sequen | *Beauveria bassiana* | 1014 | 1014 | 100% | 0.0 | 100.00% | 562 | MN947543.1 | |
| *Beauveria bassiana* isolate SASRI C2 18S ribosomal RNA gene, partial sequence | *Beauveria bassiana* | 1014 | 1014 | 100% | 0.0 | 100.00% | 594 | JX110371.1 | |
| *Cordyceps bassiana* internal transcribed spacer 1, partial sequence | *Beauveria bassiana* | 1014 | 1014 | 100% | 0.0 | 100.00% | 579 | EF672309.1 | |
| *Beauveria bassiana* isolate 1.15 small subunit ribosomal RNA gene, partial sequence | *Beauveria bassiana* | 1014 | 1014 | 100% | 0.0 | 100.00% | 551 | MH233337.1 | |
| *Beauveria bassiana* strain Ya1 small subunit ribosomal RNA gene, partial sequence | *Beauveria bassiana* | 1014 | 1014 | 100% | 0.0 | 100.00% | 593 | MK418845.1 | |
| *Beauveria bassiana* isolate strain RGM 565 small subunit ribosomal RNA gene, partial sequence | *Beauveria bassiana* | 1014 | 1014 | 100% | 0.0 | 100.00% | 563 | MH231198.1 | |
| *Beauveria bassiana* isolate B13 small subunit ribosomal RNA gene, partial sequence | *Beauveria bassiana* | 1014 | 1014 | 100% | 0.0 | 100.00% | 585 | MH922794.1 | |
| *Beauveria bassiana* genomic DNA containing ITS1, 5.8S rRNA and ITS2, strain Mas14 | *Beauveria bassiana* | 1014 | 1014 | 100% | 0.0 | 100.00% | 579 | LN823583.1 | |
| *Beauveria bassiana* strain B-Bug small subunit ribosomal RNA gene, partial sequence | *Beauveria bassiana* | 1014 | 1014 | 100% | 0.0 | 100.00% | 638 | MK862359.1 | |
| *Beauveria bassiana* isolate 1.24 small subunit ribosomal RNA gene, partial sequence | *Beauveria bassiana* | 1014 | 1014 | 100% | 0.0 | 100.00% | 559 | MH233346.1 | |
|  | | | | | | | | | |
| **Isolate 23** | | | | | | | | | |
| *Beauveria bassiana* isolate BRM068904 small subunit ribosomal RNA gene, partial sequence | *Beauveria bassiana* | 1014 | 1014 | 100% | 0.0 | 100.00% | 549 | PP506018.1 | |
| *Beauveria bassiana* strain UFRA_Bb02 small subunit ribosomal RNA gene, partial sequen | *Beauveria bassiana* | 1014 | 1014 | 100% | 0.0 | 100.00% | 562 | MN947543.1 | |
| *Beauveria bassiana* isolate SASRI C2 18S ribosomal RNA gene, partial sequence | *Beauveria bassiana* | 1014 | 1014 | 100% | 0.0 | 100.00% | 594 | JX110371.1 | |
| *Cordyceps bassiana* internal transcribed spacer 1, partial sequence | *Beauveria bassiana* | 1014 | 1014 | 100% | 0.0 | 100.00% | 579 | EF672309.1 | |
| *Beauveria bassiana* isolate 1.15 small subunit ribosomal RNA gene, partial sequence | *Beauveria bassiana* | 1014 | 1014 | 100% | 0.0 | 100.00% | 551 | MH233337.1 | |
| *Beauveria bassiana* strain Ya1 small subunit ribosomal RNA gene, partial sequence | *Beauveria bassiana* | 1014 | 1014 | 100% | 0.0 | 100.00% | 593 | MK418845.1 | |
| *Beauveria bassiana* isolate strain RGM 565 small subunit ribosomal RNA gene, partial sequence | *Beauveria bassiana* | 1014 | 1014 | 100% | 0.0 | 100.00% | 563 | MH231198.1 | |
| *Beauveria bassiana* isolate B13 small subunit ribosomal RNA gene, partial sequence | *Beauveria bassiana* | 1014 | 1014 | 100% | 0.0 | 100.00% | 585 | MH922794.1 | |
| *Beauveria bassiana* genomic DNA containing ITS1, 5.8S rRNA and ITS2, strain Mas14 | *Beauveria bassiana* | 1014 | 1014 | 100% | 0.0 | 100.00% | 579 | LN823583.1 | |
| *Beauveria bassiana* strain B-Bug small subunit ribosomal RNA gene, partial sequence | *Beauveria bassiana* | 1014 | 1014 | 100% | 0.0 | 100.00% | 638 | MK862359.1 | |
|  | | | | | | | | | |
| ***Beauveria* identification – *B-TUB* gene** | | | | | | | | | |
| **Isolate 12** | | | | | | | | | |
| [*Beauveria bassiana* isolate CG1481 beta-tubulin (TUB) gene, partial cds](https://blast.ncbi.nlm.nih.gov/Blast.cgi#alnHdr_2713927025) | [*Beauveria bassiana*](https://www.ncbi.nlm.nih.gov/Taxonomy/Browser/wwwtax.cgi?id=176275) | 678 | 678 | 99% | 0.0 | 99.47% | 375 | [PP598663.1](https://www.ncbi.nlm.nih.gov/nucleotide/PP598663.1?report=genbank&log$=nucltop&blast_rank=1&RID=R2AJ36KX014) | |
| [*Beauveria bassiana* isolate CG1478 beta-tubulin (TUB) gene, partial cds](https://blast.ncbi.nlm.nih.gov/Blast.cgi#alnHdr_2713927033) | [*Beauveria bassiana*](https://www.ncbi.nlm.nih.gov/Taxonomy/Browser/wwwtax.cgi?id=176275) | 678 | 678 | 97% | 0.0 | 100.00% | 367 | [PP598667.1](https://www.ncbi.nlm.nih.gov/nucleotide/PP598667.1?report=genbank&log$=nucltop&blast_rank=2&RID=R2AJ36KX014) | |
| [*Beauveria bassiana* isolate CG1480 beta-tubulin (TUB) gene, partial cds](https://blast.ncbi.nlm.nih.gov/Blast.cgi#alnHdr_2713927031) | [*Beauveria bassiana*](https://www.ncbi.nlm.nih.gov/Taxonomy/Browser/wwwtax.cgi?id=176275) | 676 | 676 | 99% | 0.0 | 99.20% | 378 | [PP598666.1](https://www.ncbi.nlm.nih.gov/nucleotide/PP598666.1?report=genbank&log$=nucltop&blast_rank=3&RID=R2AJ36KX014) | |
| [*Beauveria bassiana* isolate CG1479 beta-tubulin (TUB) gene, partial cds](https://blast.ncbi.nlm.nih.gov/Blast.cgi#alnHdr_2713927027) | [*Beauveria bassiana*](https://www.ncbi.nlm.nih.gov/Taxonomy/Browser/wwwtax.cgi?id=176275) | 671 | 671 | 97% | 0.0 | 99.73% | 366 | [PP598664.1](https://www.ncbi.nlm.nih.gov/nucleotide/PP598664.1?report=genbank&log$=nucltop&blast_rank=4&RID=R2AJ36KX014) | |
| [*Beauveria bassiana* isolate CG1477 beta-tubulin (TUB) gene, partial cds](https://blast.ncbi.nlm.nih.gov/Blast.cgi#alnHdr_2713927029) | [*Beauveria bassiana*](https://www.ncbi.nlm.nih.gov/Taxonomy/Browser/wwwtax.cgi?id=176275) | 665 | 665 | 99% | 0.0 | 98.93% | 380 | [PP598665.1](https://www.ncbi.nlm.nih.gov/nucleotide/PP598665.1?report=genbank&log$=nucltop&blast_rank=5&RID=R2AJ36KX014) | |
| *Beauveria bassiana* partial tub1 gene for beta tubulin, exons 1-5 | [*Beauveria bassiana*](https://www.ncbi.nlm.nih.gov/Taxonomy/Browser/wwwtax.cgi?id=176275) | 647 | 647 | 95% | 0.0 | 99.44% | 1730 | [AJ312228.1](https://www.ncbi.nlm.nih.gov/nucleotide/AJ312228.1?report=genbank&log$=nucltop&blast_rank=6&RID=R2AJ36KX014) | |
| [*Beauveria bassiana* isolate ZXYBb2 beta-tubulin 2 gene, partial cds](https://blast.ncbi.nlm.nih.gov/Blast.cgi#alnHdr_2515612339) | [*Beauveria bassiana*](https://www.ncbi.nlm.nih.gov/Taxonomy/Browser/wwwtax.cgi?id=176275) | 647 | 647 | 95% | 0.0 | 99.44% | 661 | [ON994422.1](https://www.ncbi.nlm.nih.gov/nucleotide/ON994422.1?report=genbank&log$=nucltop&blast_rank=7&RID=R2AJ36KX014) | |
| *Beauveria bassiana* isolate ZXYBb1 beta-tubulin 2 gene, partial cds | [*Beauveria bassiana*](https://www.ncbi.nlm.nih.gov/Taxonomy/Browser/wwwtax.cgi?id=176275) | 647 | 647 | 95% | 0.0 | 99.44% | 661 | [ON994421.1](https://www.ncbi.nlm.nih.gov/nucleotide/ON994421.1?report=genbank&log$=nucltop&blast_rank=8&RID=R2AJ36KX014) | |
| [*Beauveria bassiana* strain IIHRBb7 beta-tubulin gene, partial cds](https://blast.ncbi.nlm.nih.gov/Blast.cgi#alnHdr_2973164765) | [*Beauveria bassiana*](https://www.ncbi.nlm.nih.gov/Taxonomy/Browser/wwwtax.cgi?id=176275) | 627 | 627 | 90% | 6e-175 | 100.00% | 537 | [PV614324.1](https://www.ncbi.nlm.nih.gov/nucleotide/PV614324.1?report=genbank&log$=nucltop&blast_rank=9&RID=R2AJ36KX014) | |
| *Beauveria bassiana strain IIHRBb8 beta-tubulin gene, partial cds* | [*Beauveria bassiana*](https://www.ncbi.nlm.nih.gov/Taxonomy/Browser/wwwtax.cgi?id=176275) | 595 | 595 | 85% | 2e-165 | 100.00% | 515 | [PV614325.1](https://www.ncbi.nlm.nih.gov/nucleotide/PV614325.1?report=genbank&log$=nucltop&blast_rank=10&RID=R2AJ36KX014) | |
|  | | | | | | | | | |
| **Isolate 17** | | | | | | | | | |
| [*Beauveria bassiana* isolate CG1477 beta-tubulin (TUB) gene, partial cds](https://blast.ncbi.nlm.nih.gov/Blast.cgi#alnHdr_2713927029) | [*Beauveria bassiana*](https://www.ncbi.nlm.nih.gov/Taxonomy/Browser/wwwtax.cgi?id=176275) | 702 | 702 | 100% | 0.0 | 100.00% | 380 | [PP598665.1](https://www.ncbi.nlm.nih.gov/nucleotide/PP598665.1?report=genbank&log$=nucltop&blast_rank=1&RID=R2BNUA3V014) | |
| [*Beauveria bassiana* isolate CG1480 beta-tubulin (TUB) gene, partial cds](https://blast.ncbi.nlm.nih.gov/Blast.cgi#alnHdr_2713927031) | [*Beauveria bassiana*](https://www.ncbi.nlm.nih.gov/Taxonomy/Browser/wwwtax.cgi?id=176275) | 678 | 678 | 99% | 0.0 | 99.20% | 378 | [PP598666.1](https://www.ncbi.nlm.nih.gov/nucleotide/PP598666.1?report=genbank&log$=nucltop&blast_rank=2&RID=R2BNUA3V014) | |
| [*Beauveria bassiana* isolate CG1481 beta-tubulin (TUB) gene, partial cds](https://blast.ncbi.nlm.nih.gov/Blast.cgi#alnHdr_2713927025) | [*Beauveria bassiana*](https://www.ncbi.nlm.nih.gov/Taxonomy/Browser/wwwtax.cgi?id=176275) | 675 | 675 | 98% | 0.0 | 99.46% | 375 | [PP598663.1](https://www.ncbi.nlm.nih.gov/nucleotide/PP598663.1?report=genbank&log$=nucltop&blast_rank=3&RID=R2BNUA3V014) | |
| [*Beauveria bassiana* isolate CG1478 beta-tubulin (TUB) gene, partial cds](https://blast.ncbi.nlm.nih.gov/Blast.cgi#alnHdr_2713927033) | [*Beauveria bassiana*](https://www.ncbi.nlm.nih.gov/Taxonomy/Browser/wwwtax.cgi?id=176275) | 660 | 660 | 96% | 0.0 | 99.45% | 367 | [PP598667.1](https://www.ncbi.nlm.nih.gov/nucleotide/PP598667.1?report=genbank&log$=nucltop&blast_rank=4&RID=R2BNUA3V014) | |
| [*Beauveria bassiana* isolate CG1479 beta-tubulin (TUB) gene, partial cds](https://blast.ncbi.nlm.nih.gov/Blast.cgi#alnHdr_2713927027) | [*Beauveria bassiana*](https://www.ncbi.nlm.nih.gov/Taxonomy/Browser/wwwtax.cgi?id=176275) | 654 | 654 | 96% | 0.0 | 99.18% | 366 | [PP598664.1](https://www.ncbi.nlm.nih.gov/nucleotide/PP598664.1?report=genbank&log$=nucltop&blast_rank=5&RID=R2BNUA3V014) | |
| *Beauveria bassiana* partial tub1 gene for beta tubulin, exons 1-5 | [*Beauveria bassiana*](https://www.ncbi.nlm.nih.gov/Taxonomy/Browser/wwwtax.cgi?id=176275) | 647 | 647 | 94% | 0.0 | 99.44% | 1730 | [AJ312228.1](https://www.ncbi.nlm.nih.gov/nucleotide/AJ312228.1?report=genbank&log$=nucltop&blast_rank=6&RID=R2BNUA3V014) | |
| [*Beauveria bassiana* isolate ZXYBb2 beta-tubulin 2 gene, partial cds](https://blast.ncbi.nlm.nih.gov/Blast.cgi#alnHdr_2515612339) | [*Beauveria bassiana*](https://www.ncbi.nlm.nih.gov/Taxonomy/Browser/wwwtax.cgi?id=176275) | 647 | 647 | 94% | 0.0 | 99.44% | 661 | [ON994422.1](https://www.ncbi.nlm.nih.gov/nucleotide/ON994422.1?report=genbank&log$=nucltop&blast_rank=7&RID=R2BNUA3V014) | |
| *Beauveria bassiana* isolate ZXYBb1 beta-tubulin 2 gene, partial cds | [*Beauveria bassiana*](https://www.ncbi.nlm.nih.gov/Taxonomy/Browser/wwwtax.cgi?id=176275) | 647 | 647 | 94% | 0.0 | 99.44% | 661 | [ON994421.1](https://www.ncbi.nlm.nih.gov/nucleotide/ON994421.1?report=genbank&log$=nucltop&blast_rank=8&RID=R2BNUA3V014) | |
| [*Beauveria bassiana* strain IIHRBb7 beta-tubulin gene, partial cds](https://blast.ncbi.nlm.nih.gov/Blast.cgi#alnHdr_2973164765) | [*Beauveria bassiana*](https://www.ncbi.nlm.nih.gov/Taxonomy/Browser/wwwtax.cgi?id=176275) | 627 | 627 | 89% | 6e-175 | 100.00% | 537 | [PV614324.1](https://www.ncbi.nlm.nih.gov/nucleotide/PV614324.1?report=genbank&log$=nucltop&blast_rank=9&RID=R2BNUA3V014) | |
| *Beauveria bassiana strain IIHRBb8 beta-tubulin gene, partial cds* | [*Beauveria bassiana*](https://www.ncbi.nlm.nih.gov/Taxonomy/Browser/wwwtax.cgi?id=176275) | 595 | 595 | 85% | 2e-165 | 100.00% | 515 | [PV614325.1](https://www.ncbi.nlm.nih.gov/nucleotide/PV614325.1?report=genbank&log$=nucltop&blast_rank=10&RID=R2BNUA3V014) | |
|  | | | | | | | | | |
| **Isolate 18** | | | | | | | | | |
| [*Beauveria bassiana* isolate CG1481 beta-tubulin (TUB) gene, partial cds](https://blast.ncbi.nlm.nih.gov/Blast.cgi#alnHdr_2713927025) | [*Beauveria bassiana*](https://www.ncbi.nlm.nih.gov/Taxonomy/Browser/wwwtax.cgi?id=176275) | 693 | 693 | 100% | 0.0 | 100.00% | 375 | [PP598663.1](https://www.ncbi.nlm.nih.gov/nucleotide/PP598663.1?report=genbank&log$=nucltop&blast_rank=1&RID=R2C16PVT016) | |
| [*Beauveria bassiana* isolate CG1480 beta-tubulin (TUB) gene, partial cds](https://blast.ncbi.nlm.nih.gov/Blast.cgi#alnHdr_2713927031) | [*Beauveria bassiana*](https://www.ncbi.nlm.nih.gov/Taxonomy/Browser/wwwtax.cgi?id=176275) | 684 | 684 | 99% | 0.0 | 100.00% | 378 | [PP598666.1](https://www.ncbi.nlm.nih.gov/nucleotide/PP598666.1?report=genbank&log$=nucltop&blast_rank=2&RID=R2C16PVT016) | |
| [*Beauveria bassiana* isolate CG1477 beta-tubulin (TUB) gene, partial cds](https://blast.ncbi.nlm.nih.gov/Blast.cgi#alnHdr_2713927029) | [*Beauveria bassiana*](https://www.ncbi.nlm.nih.gov/Taxonomy/Browser/wwwtax.cgi?id=176275) | 675 | 675 | 99% | 0.0 | 99.46% | 380 | [PP598665.1](https://www.ncbi.nlm.nih.gov/nucleotide/PP598665.1?report=genbank&log$=nucltop&blast_rank=3&RID=R2C16PVT016) | |
| [*Beauveria bassiana* isolate CG1478 beta-tubulin (TUB) gene, partial cds](https://blast.ncbi.nlm.nih.gov/Blast.cgi#alnHdr_2713927033) | [*Beauveria bassiana*](https://www.ncbi.nlm.nih.gov/Taxonomy/Browser/wwwtax.cgi?id=176275) | 673 | 673 | 97% | 0.0 | 100.00% | 367 | [PP598667.1](https://www.ncbi.nlm.nih.gov/nucleotide/PP598667.1?report=genbank&log$=nucltop&blast_rank=4&RID=R2C16PVT016) | |
| [*Beauveria bassiana* isolate CG1479 beta-tubulin (TUB) gene, partial cds](https://blast.ncbi.nlm.nih.gov/Blast.cgi#alnHdr_2713927027) | [*Beauveria bassiana*](https://www.ncbi.nlm.nih.gov/Taxonomy/Browser/wwwtax.cgi?id=176275) | 665 | 665 | 97% | 0.0 | 99.73% | 366 | [PP598664.1](https://www.ncbi.nlm.nih.gov/nucleotide/PP598664.1?report=genbank&log$=nucltop&blast_rank=5&RID=R2C16PVT016) | |
| *Beauveria bassiana* partial tub1 gene for beta tubulin, exons 1-5 | [*Beauveria bassiana*](https://www.ncbi.nlm.nih.gov/Taxonomy/Browser/wwwtax.cgi?id=176275) | 647 | 647 | 95% | 0.0 | 99.44% | 1730 | [AJ312228.1](https://www.ncbi.nlm.nih.gov/nucleotide/AJ312228.1?report=genbank&log$=nucltop&blast_rank=6&RID=R2C16PVT016) | |
| [*Beauveria bassiana* isolate ZXYBb2 beta-tubulin 2 gene, partial cds](https://blast.ncbi.nlm.nih.gov/Blast.cgi#alnHdr_2515612339) | [*Beauveria bassiana*](https://www.ncbi.nlm.nih.gov/Taxonomy/Browser/wwwtax.cgi?id=176275) | 647 | 647 | 95% | 0.0 | 99.44% | 661 | [ON994422.1](https://www.ncbi.nlm.nih.gov/nucleotide/ON994422.1?report=genbank&log$=nucltop&blast_rank=7&RID=R2C16PVT016) | |
| *Beauveria bassiana* isolate ZXYBb1 beta-tubulin 2 gene, partial cds | [*Beauveria bassiana*](https://www.ncbi.nlm.nih.gov/Taxonomy/Browser/wwwtax.cgi?id=176275) | 647 | 647 | 95% | 0.0 | 99.44% | 661 | [ON994421.1](https://www.ncbi.nlm.nih.gov/nucleotide/ON994421.1?report=genbank&log$=nucltop&blast_rank=8&RID=R2C16PVT016) | |
| [*Beauveria bassiana* strain IIHRBb7 beta-tubulin gene, partial cds](https://blast.ncbi.nlm.nih.gov/Blast.cgi#alnHdr_2973164765) | [*Beauveria bassiana*](https://www.ncbi.nlm.nih.gov/Taxonomy/Browser/wwwtax.cgi?id=176275) | 627 | 627 | 90% | 6e-175 | 100.00% | 537 | [PV614324.1](https://www.ncbi.nlm.nih.gov/nucleotide/PV614324.1?report=genbank&log$=nucltop&blast_rank=9&RID=R2C16PVT016) | |
| *Beauveria bassiana strain IIHRBb8 beta-tubulin gene, partial cds* | [*Beauveria bassiana*](https://www.ncbi.nlm.nih.gov/Taxonomy/Browser/wwwtax.cgi?id=176275) | 595 | 595 | 86% | 2e-165 | 100.00% | 515 | [PV614325.1](https://www.ncbi.nlm.nih.gov/nucleotide/PV614325.1?report=genbank&log$=nucltop&blast_rank=10&RID=R2C16PVT016) | |
|  | | | | | | | | | |
| **Isolate 19** | | | | | | | | | |
| [*Beauveria bassiana* isolate CG1480 beta-tubulin (TUB) gene, partial cds](https://blast.ncbi.nlm.nih.gov/Blast.cgi#alnHdr_2713927031) | [*Beauveria bassiana*](https://www.ncbi.nlm.nih.gov/Taxonomy/Browser/wwwtax.cgi?id=176275) | 699 | 699 | 100% | 0.0 | 100.00% | 378 | [PP598666.1](https://www.ncbi.nlm.nih.gov/nucleotide/PP598666.1?report=genbank&log$=nucltop&blast_rank=1&RID=R2CEGDDR014) | |
| [*Beauveria bassiana* isolate CG1481 beta-tubulin (TUB) gene, partial cds](https://blast.ncbi.nlm.nih.gov/Blast.cgi#alnHdr_2713927025) | [*Beauveria bassiana*](https://www.ncbi.nlm.nih.gov/Taxonomy/Browser/wwwtax.cgi?id=176275) | 684 | 684 | 98% | 0.0 | 100.00% | 375 | [PP598663.1](https://www.ncbi.nlm.nih.gov/nucleotide/PP598663.1?report=genbank&log$=nucltop&blast_rank=2&RID=R2CEGDDR014) | |
| [*Beauveria bassiana* isolate CG1477 beta-tubulin (TUB) gene, partial cds](https://blast.ncbi.nlm.nih.gov/Blast.cgi#alnHdr_2713927029) | [*Beauveria bassiana*](https://www.ncbi.nlm.nih.gov/Taxonomy/Browser/wwwtax.cgi?id=176275) | 678 | 678 | 99% | 0.0 | 99.20% | 380 | [PP598665.1](https://www.ncbi.nlm.nih.gov/nucleotide/PP598665.1?report=genbank&log$=nucltop&blast_rank=3&RID=R2CEGDDR014) | |
| [*Beauveria bassiana* isolate CG1478 beta-tubulin (TUB) gene, partial cds](https://blast.ncbi.nlm.nih.gov/Blast.cgi#alnHdr_2713927033) | [*Beauveria bassiana*](https://www.ncbi.nlm.nih.gov/Taxonomy/Browser/wwwtax.cgi?id=176275) | 673 | 673 | 97% | 0.0 | 99.73% | 367 | [PP598667.1](https://www.ncbi.nlm.nih.gov/nucleotide/PP598667.1?report=genbank&log$=nucltop&blast_rank=4&RID=R2CEGDDR014) | |
| [*Beauveria bassiana* isolate CG1479 beta-tubulin (TUB) gene, partial cds](https://blast.ncbi.nlm.nih.gov/Blast.cgi#alnHdr_2713927027) | [*Beauveria bassiana*](https://www.ncbi.nlm.nih.gov/Taxonomy/Browser/wwwtax.cgi?id=176275) | 667 | 667 | 97% | 0.0 | 99.46% | 366 | [PP598664.1](https://www.ncbi.nlm.nih.gov/nucleotide/PP598664.1?report=genbank&log$=nucltop&blast_rank=5&RID=R2CEGDDR014) | |
| *Beauveria bassiana* partial tub1 gene for beta tubulin, exons 1-5 | [*Beauveria bassiana*](https://www.ncbi.nlm.nih.gov/Taxonomy/Browser/wwwtax.cgi?id=176275) | 647 | 647 | 94% | 0.0 | 99.44% | 1730 | [AJ312228.1](https://www.ncbi.nlm.nih.gov/nucleotide/AJ312228.1?report=genbank&log$=nucltop&blast_rank=6&RID=R2CEGDDR014) | |
| [*Beauveria bassiana* isolate ZXYBb2 beta-tubulin 2 gene, partial cds](https://blast.ncbi.nlm.nih.gov/Blast.cgi#alnHdr_2515612339) | [*Beauveria bassiana*](https://www.ncbi.nlm.nih.gov/Taxonomy/Browser/wwwtax.cgi?id=176275) | 647 | 647 | 94% | 0.0 | 99.44% | 661 | [ON994422.1](https://www.ncbi.nlm.nih.gov/nucleotide/ON994422.1?report=genbank&log$=nucltop&blast_rank=7&RID=R2CEGDDR014) | |
| *Beauveria bassiana* isolate ZXYBb1 beta-tubulin 2 gene, partial cds | [*Beauveria bassiana*](https://www.ncbi.nlm.nih.gov/Taxonomy/Browser/wwwtax.cgi?id=176275) | 647 | 647 | 94% | 0.0 | 99.44% | 661 | [ON994421.1](https://www.ncbi.nlm.nih.gov/nucleotide/ON994421.1?report=genbank&log$=nucltop&blast_rank=8&RID=R2CEGDDR014) | |
| [*Beauveria bassiana* strain IIHRBb7 beta-tubulin gene, partial cds](https://blast.ncbi.nlm.nih.gov/Blast.cgi#alnHdr_2973164765) | [*Beauveria bassiana*](https://www.ncbi.nlm.nih.gov/Taxonomy/Browser/wwwtax.cgi?id=176275) | 627 | 627 | 90% | 6e-175 | 100.00% | 537 | [PV614324.1](https://www.ncbi.nlm.nih.gov/nucleotide/PV614324.1?report=genbank&log$=nucltop&blast_rank=9&RID=R2CEGDDR014) | |
| *Beauveria bassiana strain IIHRBb8 beta-tubulin gene, partial cds* | [*Beauveria bassiana*](https://www.ncbi.nlm.nih.gov/Taxonomy/Browser/wwwtax.cgi?id=176275) | 595 | 595 | 85% | 2e-165 | 100.00% | 515 | [PV614325.1](https://www.ncbi.nlm.nih.gov/nucleotide/PV614325.1?report=genbank&log$=nucltop&blast_rank=10&RID=R2CEGDDR014) | |
|  | | | | | | | | | |
| **Isolate 23** | | | | | | | | | |
| [*Beauveria bassiana* isolate CG1479 beta-tubulin (TUB) gene, partial cds](https://blast.ncbi.nlm.nih.gov/Blast.cgi#alnHdr_2713927027) | [*Beauveria bassiana*](https://www.ncbi.nlm.nih.gov/Taxonomy/Browser/wwwtax.cgi?id=176275) | 676 | 676 | 98% | 0.0 | 100.00% | 366 | [PP598664.1](https://www.ncbi.nlm.nih.gov/nucleotide/PP598664.1?report=genbank&log$=nucltop&blast_rank=1&RID=R2CS7A04014) | |
| [*Beauveria bassiana* isolate CG1480 beta-tubulin (TUB) gene, partial cds](https://blast.ncbi.nlm.nih.gov/Blast.cgi#alnHdr_2713927031) | [*Beauveria bassiana*](https://www.ncbi.nlm.nih.gov/Taxonomy/Browser/wwwtax.cgi?id=176275) | 671 | 671 | 100% | 0.0 | 98.94% | 378 | [PP598666.1](https://www.ncbi.nlm.nih.gov/nucleotide/PP598666.1?report=genbank&log$=nucltop&blast_rank=2&RID=R2CS7A04014) | |
| [*Beauveria bassiana* isolate CG1478 beta-tubulin (TUB) gene, partial cds](https://blast.ncbi.nlm.nih.gov/Blast.cgi#alnHdr_2713927033) | [*Beauveria bassiana*](https://www.ncbi.nlm.nih.gov/Taxonomy/Browser/wwwtax.cgi?id=176275) | 671 | 671 | 98% | 0.0 | 99.73% | 367 | [PP598667.1](https://www.ncbi.nlm.nih.gov/nucleotide/PP598667.1?report=genbank&log$=nucltop&blast_rank=3&RID=R2CS7A04014) | |
| [*Beauveria bassiana* isolate CG1481 beta-tubulin (TUB) gene, partial cds](https://blast.ncbi.nlm.nih.gov/Blast.cgi#alnHdr_2713927025) | [*Beauveria bassiana*](https://www.ncbi.nlm.nih.gov/Taxonomy/Browser/wwwtax.cgi?id=176275) | 669 | 669 | 99% | 0.0 | 99.19% | 375 | [PP598663.1](https://www.ncbi.nlm.nih.gov/nucleotide/PP598663.1?report=genbank&log$=nucltop&blast_rank=4&RID=R2CS7A04014) | |
| *Beauveria bassiana* isolate CG1477 beta-tubulin (TUB) gene, partial cds | [*Beauveria bassiana*](https://www.ncbi.nlm.nih.gov/Taxonomy/Browser/wwwtax.cgi?id=176275) | 656 | 656 | 99% | 0.0 | 98.66% | 380 | [PP598665.1](https://www.ncbi.nlm.nih.gov/nucleotide/PP598665.1?report=genbank&log$=nucltop&blast_rank=5&RID=R2CS7A04014) | |
| *Beauveria bassiana* partial tub1 gene for beta tubulin, exons 1-5 | [*Beauveria bassiana*](https://www.ncbi.nlm.nih.gov/Taxonomy/Browser/wwwtax.cgi?id=176275) | 647 | 647 | 95% | 0.0 | 99.44% | 1730 | [AJ312228.1](https://www.ncbi.nlm.nih.gov/nucleotide/AJ312228.1?report=genbank&log$=nucltop&blast_rank=6&RID=R2CS7A04014) | |
| [*Beauveria bassiana* isolate ZXYBb2 beta-tubulin 2 gene, partial cds](https://blast.ncbi.nlm.nih.gov/Blast.cgi#alnHdr_2515612339) | [*Beauveria bassiana*](https://www.ncbi.nlm.nih.gov/Taxonomy/Browser/wwwtax.cgi?id=176275) | 647 | 647 | 95% | 0.0 | 99.44% | 661 | [ON994422.1](https://www.ncbi.nlm.nih.gov/nucleotide/ON994422.1?report=genbank&log$=nucltop&blast_rank=7&RID=R2CS7A04014) | |
| *Beauveria bassiana* isolate ZXYBb1 beta-tubulin 2 gene, partial cds | [*Beauveria bassiana*](https://www.ncbi.nlm.nih.gov/Taxonomy/Browser/wwwtax.cgi?id=176275) | 647 | 647 | 95% | 0.0 | 99.44% | 661 | [ON994421.1](https://www.ncbi.nlm.nih.gov/nucleotide/ON994421.1?report=genbank&log$=nucltop&blast_rank=8&RID=R2CS7A04014) | |
| [*Beauveria bassiana* strain IIHRBb7 beta-tubulin gene, partial cds](https://blast.ncbi.nlm.nih.gov/Blast.cgi#alnHdr_2973164765) | [*Beauveria bassiana*](https://www.ncbi.nlm.nih.gov/Taxonomy/Browser/wwwtax.cgi?id=176275) | 627 | 627 | 90% | 6e-175 | 100.00% | 537 | [PV614324.1](https://www.ncbi.nlm.nih.gov/nucleotide/PV614324.1?report=genbank&log$=nucltop&blast_rank=9&RID=R2CS7A04014) | |
| *Beauveria bassiana strain IIHRBb8 beta-tubulin gene, partial cds* | [*Beauveria bassiana*](https://www.ncbi.nlm.nih.gov/Taxonomy/Browser/wwwtax.cgi?id=176275) | 595 | 595 | 86% | 2e-165 | 100.00% | 515 | [PV614325.1](https://www.ncbi.nlm.nih.gov/nucleotide/PV614325.1?report=genbank&log$=nucltop&blast_rank=10&RID=R2CS7A04014) | |
|  | | | | | | | | | |
| ***Metarhizium* identification – *TEF*-1 gene** | | | | | | | | |  |
| **Isolate 1** | | | | | | | | |  |
| *Metarhizium pingshaense* isolate CG1476 translation elongation factor 1-alpha (TEF) gene, partial cds | *Metarhizium pinghaense* | 1423 | 1423 | 98% | 0.0 | 100.00% | 770 | PP590613.1 | |
| *Metarhizium pingshaense* strain ARSEF 3210 translation elongation factor 1 alpha (EF1alpha) gene, partial cds | *Metarhizium pinghaense* | 1375 | 1375 | 97% | 0.0 | 99.34% | 1717 | DQ463995.1 | |
| *Metarhizium pinghaense* culture-collection ARSEF:7929 translation elongation factor 1 alpha (TEF) gene, partial sequence | *Metarhizium pinghaense* | 1371 | 1371 | 97% | 0.0 | 99.21% | 1706 | EU248847.1 | |
| *Metarhizium pinghaense* culture-collection CBS:257.90 translation elongation factor 1 alpha (TEF) gene, partial sequence | *Metarhizium pinghaense* | 1371 | 1371 | 97% | 0.0 | 99.21% | 1699 | EU248850.1 | |
| *Metarhizium pinghaense* culture-collection ARSEF:4342 translation elongation factor 1 alpha (TEF) gene, partial sequence | *Metarhizium pinghaense* | 1365 | 1365 | 97% | 0.0 | 99.08% | 1718 | EU248851.1 | |
| *Metarhizium pinghaense* isolate RMT23 translation elongation factor 1-alpha (EF1alpha) gene, partial cds | *Metarhizium pinghaense* | 1354 | 1354 | 96% | 0.0 | 99.33% | 1451 | KC870072.1 | |
| *Metarhizium pinghaense* isolate MET 13/I68 translation elongation factor 1-alpha gene, partial cds | *Metarhizium pinghaense* | 1351 | 1351 | 97% | 0.0 | 98.82% | 980 | KJ588065.1 | |
| *Metarhizium robertsii* isolate DAOM 241872 translation elongation factor 1-alpha (EF1alpha) gene, partial cds | *Metarhizium robertsii* | 1345 | 1345 | 97% | 0.0 | 98.69% | 807 | MK391226.1 | |
| *Metarhizium robertsii* isolate LRC 207 translation elongation factor 1-alpha (EF1alpha) gene, partial cds | *Metarhizium robertsii* | 1345 | 1345 | 97% | 0.0 | 98.69% | 816 | MK391196.1 | |
| *Metarhizium robertsii* isolate DAOM 241898 translation elongation factor 1-alpha (EF1alpha) gene, partial cds | *Metarhizium robertsii* | 1339 | 1339 | 97% | 0.0 | 98.55% | 813 | MK391187.1 | |
| ***Metarhizium* identification – *B-TUB* gene** | | | | | | | | | |
| **Isolate 1** | | | | | | | | | |
| *Metarhizium pingshaense* isolate CG1476 beta-tubulin (TUB) gene, partial cds | [*Metarhizium pingshaense*](https://www.ncbi.nlm.nih.gov/Taxonomy/Browser/wwwtax.cgi?id=1278899) | 754 | 754 | 100% | 0.0 | 100.00% | 408 | [PP598662.1](https://www.ncbi.nlm.nih.gov/nucleotide/PP598662.1?report=genbank&log$=nucltop&blast_rank=1&RID=R2DAHGGJ016) | |
| [*Metarhizium pingshaense* beta-tubulin gene, partial cds](https://blast.ncbi.nlm.nih.gov/Blast.cgi#alnHdr_2635774177) | [*Metarhizium pingshaense*](https://www.ncbi.nlm.nih.gov/Taxonomy/Browser/wwwtax.cgi?id=1278899) | 691 | 691 | 95% | 0.0 | 98.72% | 1221 | [OR900300.1](https://www.ncbi.nlm.nih.gov/nucleotide/OR900300.1?report=genbank&log$=nucltop&blast_rank=2&RID=R2DAHGGJ016) | |
| [*Metarhizium pingshaense* isolate SG-D beta-tubulin gene, partial cds](https://blast.ncbi.nlm.nih.gov/Blast.cgi#alnHdr_2878244737) | [*Metarhizium pingshaense*](https://www.ncbi.nlm.nih.gov/Taxonomy/Browser/wwwtax.cgi?id=1278899) | 691 | 691 | 95% | 0.0 | 98.72% | 1209 | [PQ606085.1](https://www.ncbi.nlm.nih.gov/nucleotide/PQ606085.1?report=genbank&log$=nucltop&blast_rank=3&RID=R2DAHGGJ016) | |
| [*Metarhizium pinghaense* culture-collection ARSEF:4342 beta-tubulin gene, partial sequence](https://blast.ncbi.nlm.nih.gov/Blast.cgi#alnHdr_166406337) | [*Metarhizium pingshaense*](https://www.ncbi.nlm.nih.gov/Taxonomy/Browser/wwwtax.cgi?id=1278899) | 691 | 691 | 95% | 0.0 | 98.72% | 1327 | [EU248821.1](https://www.ncbi.nlm.nih.gov/nucleotide/EU248821.1?report=genbank&log$=nucltop&blast_rank=4&RID=R2DAHGGJ016) | |
| *Metarhizium pingshaense* beta-tubulin gene, partial cds | [*Metarhizium pingshaense*](https://www.ncbi.nlm.nih.gov/Taxonomy/Browser/wwwtax.cgi?id=1278899) | 691 | 691 | 95% | 0.0 | 98.72% | 1229 | [PQ110649.1](https://www.ncbi.nlm.nih.gov/nucleotide/PQ110649.1?report=genbank&log$=nucltop&blast_rank=5&RID=R2DAHGGJ016) | |
| [*Metarhizium pingshaense* isolate SG-C beta-tubulin gene, partial cds](https://blast.ncbi.nlm.nih.gov/Blast.cgi#alnHdr_2878244735) | [*Metarhizium pingshaense*](https://www.ncbi.nlm.nih.gov/Taxonomy/Browser/wwwtax.cgi?id=1278899) | 691 | 691 | 95% | 0.0 | 98.72% | 1200 | [PQ606084.1](https://www.ncbi.nlm.nih.gov/nucleotide/PQ606084.1?report=genbank&log$=nucltop&blast_rank=6&RID=R2DAHGGJ016) | |
| [*Metarhizium* sp. isolate URM8140 beta-tubulin gene, partial cds](https://blast.ncbi.nlm.nih.gov/Blast.cgi#alnHdr_2312977449) | [*Metarhizium* sp.](https://www.ncbi.nlm.nih.gov/Taxonomy/Browser/wwwtax.cgi?id=1884861) | 678 | 678 | 95% | 0.0 | 98.20% | 404 | [MZ394811.1](https://www.ncbi.nlm.nih.gov/nucleotide/MZ394811.1?report=genbank&log$=nucltop&blast_rank=7&RID=R2DAHGGJ016) | |
| [*Metarhizium* sp. isolate URM8139 beta-tubulin gene, partial cds](https://blast.ncbi.nlm.nih.gov/Blast.cgi#alnHdr_2312977447) | [*Metarhizium* sp.](https://www.ncbi.nlm.nih.gov/Taxonomy/Browser/wwwtax.cgi?id=1884861) | 678 | 678 | 95% | 0.0 | 98.20% | 404 | [MZ394810.1](https://www.ncbi.nlm.nih.gov/nucleotide/MZ394810.1?report=genbank&log$=nucltop&blast_rank=8&RID=R2DAHGGJ016) | |
| [*Metarhizium* sp. isolate URM8144 beta-tubulin gene, partial cds](https://blast.ncbi.nlm.nih.gov/Blast.cgi#alnHdr_2312977457) | [*Metarhizium* sp.](https://www.ncbi.nlm.nih.gov/Taxonomy/Browser/wwwtax.cgi?id=1884861) | 678 | 678 | 95% | 0.0 | 98.20% | 404 | [MZ394815.1](https://www.ncbi.nlm.nih.gov/nucleotide/MZ394815.1?report=genbank&log$=nucltop&blast_rank=9&RID=R2DAHGGJ016) | |
| [*Metarhizium* sp. isolate URM8142 beta-tubulin gene, partial cds](https://blast.ncbi.nlm.nih.gov/Blast.cgi#alnHdr_2312977453) | [*Metarhizium* sp.](https://www.ncbi.nlm.nih.gov/Taxonomy/Browser/wwwtax.cgi?id=1884861) | 678 | 678 | 95% | 0.0 | 98.20% | 404 | [MZ394813.1](https://www.ncbi.nlm.nih.gov/nucleotide/MZ394813.1?report=genbank&log$=nucltop&blast_rank=10&RID=R2DAHGGJ016) | |

Table S3: Codes of entomopathogenic fungal isolates and accession codes of sequenced genes deposited in NCBI.

| **Isolate** | **Specie** |  | **Isolate code** | |  | **Gene code in GenBank** | | |
| --- | --- | --- | --- | --- | --- | --- | --- | --- |
|  |  |  | **LCBiotec^1^** | **Embrapa^2^** |  | ***ITS*** | ***B-TUB*** | ***TEF-1*** |
| Isolate 1 | *Metarhizium pinghaense* |  | SE203 | BRM068839 |  | - | PP598662 | PP590613 |
| Isolate 12 | *Beauveria bassiana* |  | SE113 | BRM068907 |  | PP506014 | PP598667 | - |
| Isolate 17 | *Beauveria bassiana* |  | SE117 | BRM068905 |  | PP506015 | PP598665 | - |
| Isolate 18 | *Beauveria bassiana* |  | SE118 | BRM068840 |  | PP506016 | PP598663 | - |
| Isolate 19 | *Beauveria bassiana* |  | SE119 | BRM068906 |  | PP506017 | PP598666 | - |
| Isolate 23 | *Beauveria bassiana* |  | SE114 | BRM068904 |  | PP506018 | PP598664 | - |

**^1^** LCBiotec: Biotechnological Pest Control Laboratory – Agricultural Development Corporation of Sergipe.

**^2^** Embrapa: Brazilian Agricultural Research Corporation.

Table S4: Culture collection number and GenBank accession numbers of *ITS* region (Rehner et al. 2011; Serna-Dominguez et al. 2018) and *TEF-1* (Bischoff et al. 2009) of *Beauveria* and *Metarhizium* species sequences used to construct phylogenetic trees.

| **Specie** | **Isolate code** | **Gene code in GenBank*** |
| --- | --- | --- |
|  |  | ***ITS*** |
| *Beauveria amorpha* | ARSEF 2641 **T** | NR111601.1 |
|  | ARSEF 4149 | HQ880804.1 |
|  | ARSEF 7542 | HQ880805.1 |
| *Beauveria australis* | ARSEF 4580 | HQ880788.1 |
|  | ARSEF 4598 **T** | NR111597.1 |
| *Beauveria bassiana* | ARSEF 300 | HQ880759.1 |
|  | ARSEF 1040 | AY531972.1 |
|  | ARSEF 1478 | HQ880764.1 |
|  | ARSEF 1564 **T** | NR111594.1 |
|  | ARSEF 1811 | HQ880765.1 |
|  | ARSEF 1848 | AY531995.1 |
|  | ARSEF 7518 | HQ880762.1 |
|  | CHE-CNRCB 168 | KU725691.1 |
| *Beauveria brongniartii* | ARSEF 985 | HQ880768.1 |
|  | ARSEF 7517 | HQ880767.1 |
| *Beauveria caledonica* | ARSEF 2567 **T** | AY532006.1 |
|  | ARSEF 4302 | HQ880821.1 |
| *Beauveria varroae* | ARSEF 2694 | HQ880802.1 |
|  | ARSEF 8257 **T** | NR111599.1 |
|  |  | ***TEF-1*** |
| *Metarhizium acridum* | ARSEF 324 | EU248844.1 |
|  | ARSEF 7486 **T** | EU248845.1 |
| *Metarhizium anisopliae* | ARSEF 7450 | EU248852.1 |
|  | ARSEF 7487 **T** | DQ463996.2 |
| *Metarhizium brunneum* | ARSEF 2107 **T** | EU248855.1 |
|  | ARSEF 4152 | EU248853.1 |
|  | ARSEF 4179 | EU248854.1 |
| *Metarhizium guizhouense* | ARSEF 4303 | EU248859.1 |
|  | ARSEF 5714 | EU248856.1 |
|  | ARSEF 7502 | EU248861.1 |
|  | CBS 258.90 **T** | EU248862.1 |
| *Metarhizium majus* | ARSEF 1914 **T** | EU248868.1 |
|  | ARSEF 1946 | EU248867.1 |
|  | ARSEF 2808 | EU248871.1 |
|  | ARSEF 4566 | EU248869.1 |
|  | ARSEF 7505 | EU248870.1 |
| *Metarhizium pinghaense* | ARSEF 3210 | DQ463995.2 |
|  | ARSEF:4342 | EU248851.1 |
|  | ARSEF 7929 | EU248847.1 |
|  | CBS 257.90 **T** | EU248850.1 |

* Sequences obtained directly from the NCBI database.

**T** – Type material

**Figures**


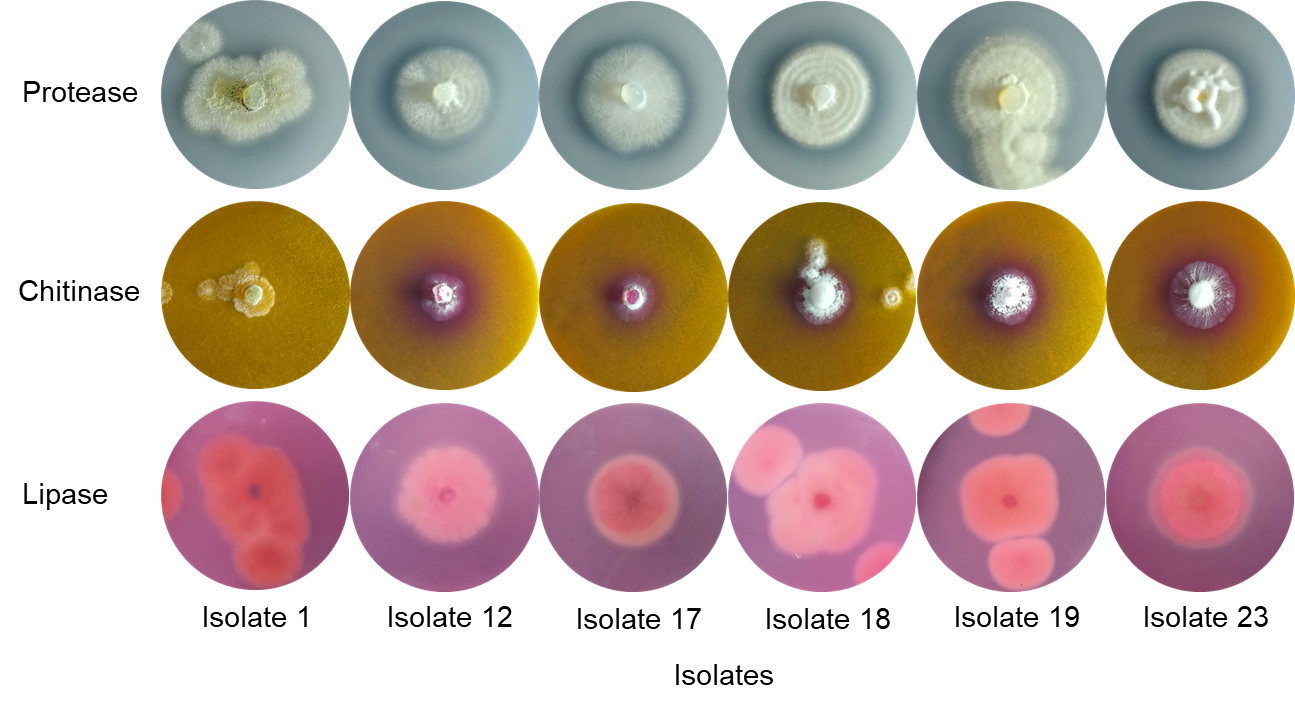


**Fig. S1** Colonies of different entomopathogenic fungal isolates grown in culture medium with inducers of protease, chitinase, and lipase enzyme activity. The presence of a clear halo and red coloration around the colonies indicates protease and chitinase enzyme activity, respectively. The presence of orange coloration, at different intensities, indicates lipase activity.


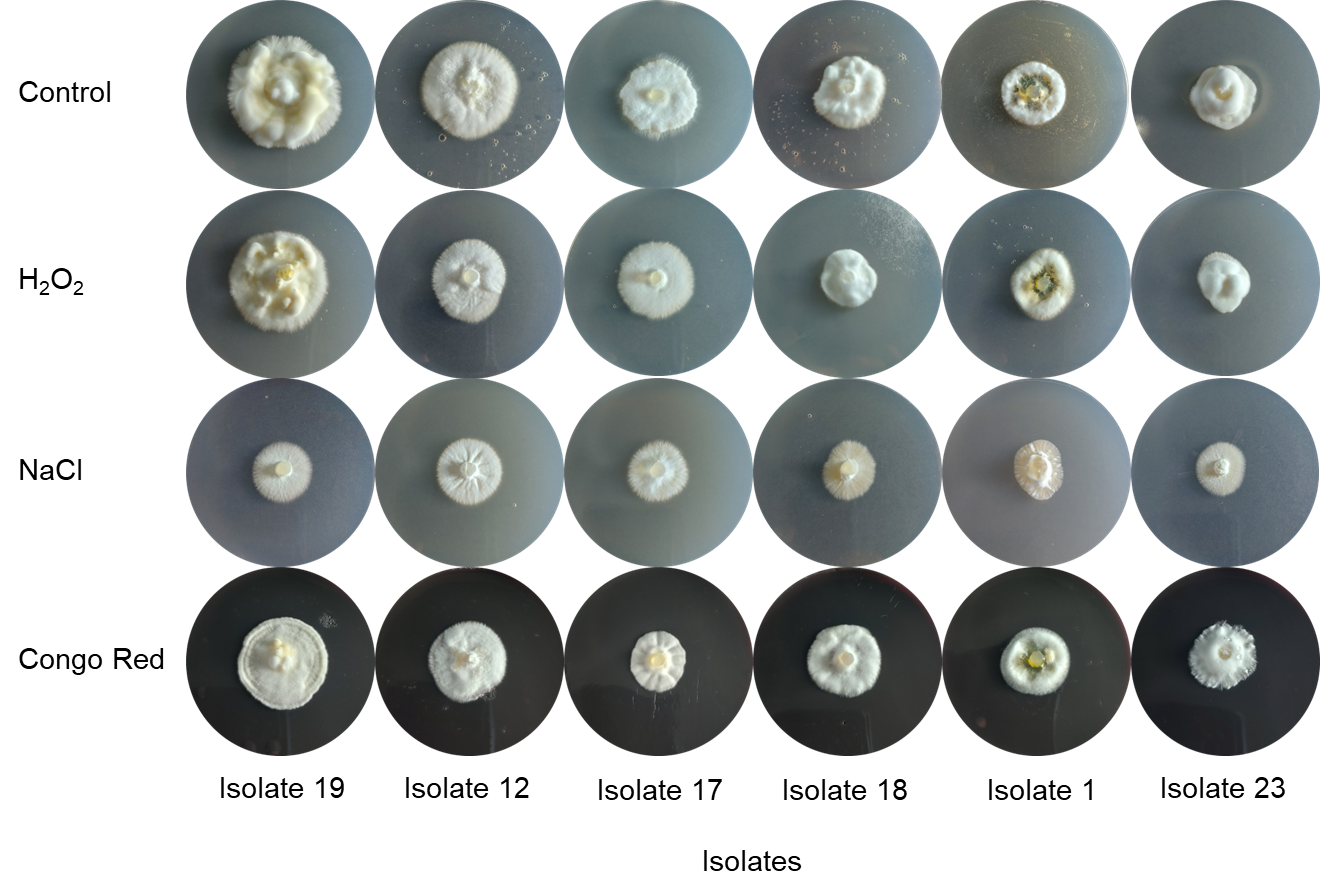


**Fig. S2** Colonies of entomopathogenic fungi grown in Petri dishes containing PDA culture medium + stress inducers.
